# Characterization and Crystal Structure Elucidation of the l‐Homoserine *O*‐Succinyltransferase MetXS From *Cupriavidus necator* H16

**DOI:** 10.1002/cbic.70564

**Published:** 2026-10-20

**Authors:** Hannah Pia Franziska Meier, Rick Oerlemans, Henrik Terholsen, Matthew R. Groves, Lars Lauterbach, Sandy Schmidt

**Affiliations:** ^1^ Department of Chemical and Pharmaceutical Biology Groningen Research Institute of Pharmacy University of Groningen Groningen The Netherlands; ^2^ iAMB ‐ Institute of Applied Microbiology RWTH Aachen University Aachen Germany

**Keywords:** acyltransferase, *Cupriavidus necator*, homoserine *O*‐succinyltransferase, methionine biosynthesis, MetX

## Abstract

Among the promising microbial platforms to produce value‐added products from CO_2_, chemolithoautotrophic bacteria, such as *Cupriavidus necator* H16 (*C. necator*), stand out due to their ability to fix CO_2_ via the Calvin–Benson–Bassham (CBB) cycle. Although *C. necator* has been extensively studied as a biotechnological production host, several of its biosynthetic pathways remain incompletely characterized at the biochemical level. This includes l‐methionine biosynthesis, for which genome‐based functional annotations of key enzymes have not been validated experimentally. In this study, we identified and characterized *Cn*MetXS, a homoserine *O*‐succinyltransferase involved in l‐methionine biosynthesis in *C. necator*. Biochemical characterization demonstrated that *Cn*MetXS catalyzes the transsuccinylation of l‐homoserine to *O*‐succinyl‐l‐homoserine but exhibits no transacetylase activity. Additionally, we determined the apo crystal structure of *Cn*MetXS at 1.86 Å resolution, revealing a homodimer with a MetX‐G2 active‐site architecture and distinct conformations of the active‐site lid region. Structural analysis and docking suggest that Arg335 stabilizes the succinylated enzyme intermediate and explains the observed succinyl‐CoA specificity. Together, these results establish the function of *Cn*MetXS and pave the way for metabolic engineering strategies to produce l‐methionine in *C. necator*.

## Graphical abstract

This study reports the identification, heterologous production, and in vitro characterization of the homoserine *O*‐succinyltransferase from *C. necator* (*Cn*MetXS). Several biochemical parameters, including the pH and temperature optima, thermostability, substrate scope, and kinetic parameters, are determined, and a high‐resolution X‐ray structure of *Cn*MetXS is obtained, revealing its role as a transsuccinylase in the l‐methionine biosynthesis in *C. necator*.

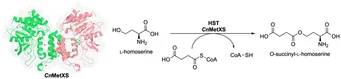

## 1 Introduction

The depletion of fossil resources has intensified efforts to replace petrochemical syntheses with biotechnological alternatives. Amino acids serve as a prime example, as most of the 20 proteinogenic amino acids are now produced by microbial fermentation using glucose as the carbon source [1, 2]. However, methionine remains exclusively synthesized via petrochemical routes [3, 4, 5]. In 2024, global methionine production reached ~1.1 million tons [6], primarily for use as a feed supplement to enhance the nutritional value of animal diets and support the growing demand for animal protein [7, 8]. Therefore, establishing sustainable fermentation‐based production of methionine is of high interest, especially considering the environmental constraints of the current chemical process [2, 9]. One promising alternative strategy is developing microbial platforms that can produce l‐methionine from CO_2_ as the primary carbon source. Using industrial off‐gas for CO_2_ supply has the potential to reduce total carbon emissions, making the production of valuable chemicals, such as amino acids, carbon‐negative or, at least, carbon‐neutral [10, 11, 12, 13], and avoiding competition with the food industry for sugars as the carbon source.

The chemolithoautotroph *Cupriavidus necator* H16 (*C. necator*, formerly known as *Ralstonia eutropha* [14]) is a promising chassis for developing such an alternative route from CO_2_ [15, 16, 17]. It is well known for its ability to accumulate polyhydroxybutyrate (PHB) [18, 19] and for its hydrogenases, which can sustain catalytic activity in the presence of O_2_ [20]. Moreover, *C. necator* has been successfully engineered to produce various value‐added products, including fatty acids [21], hydrocarbons [22], isopropanol [23, 24], and isotope‐labeled amino acids [10].

In bacteria, l‐methionine can be synthesized through two alternative routes: the trans‐sulfurylation pathway and the direct‐sulfurylation pathway [25]. Both routes converge on the transformation of l‐homoserine into l‐homocysteine, but they differ in the number of enzymatic steps and the nature of the sulfur donor. The trans‐sulfurylation pathway involves three enzymatic reactions (Scheme 1).

**SCHEME 1:**
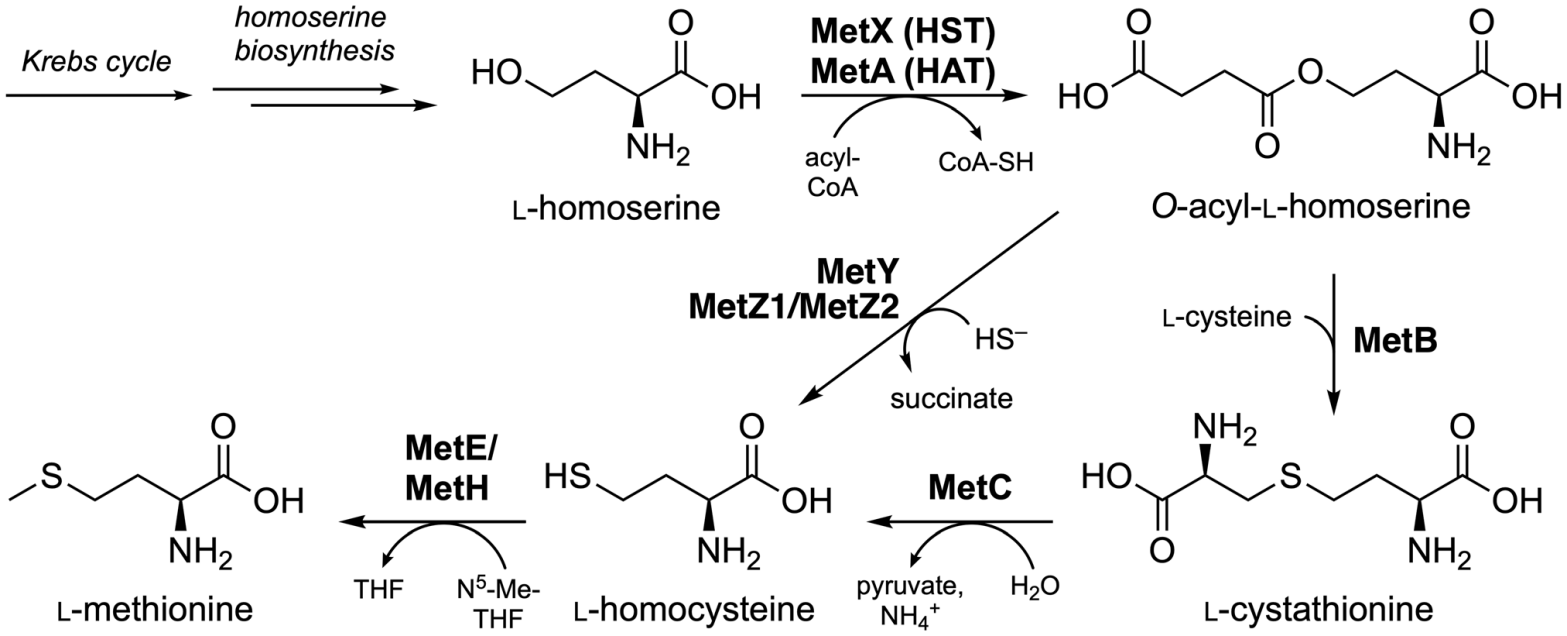
Transsulfurylation and direct‐sulfurylation l‐methionine biosynthetic pathways. l‐homoserine is derived from Krebs cycle precursors. In the transsulfurylation pathway, l‐homoserine *O*‐succinyltransferase (HST) catalyzes the formation of *O*‐succinyl‐l‐homoserine. One molecule of l‐cysteine reacts with the *O*‐succinyl‐l‐homoserine to form cystathionine catalyzed by cystathionine γ‐synthase (MetB). Cystathionine β‐lyase (MetC) cleaves cystathionine to yield l‐homocysteine. In the direct‐sulfurylation pathway, l‐homoserine *O*‐acetyltransferase (HAT) synthesizes *O*‐acetyl‐l‐homoserine from l‐homoserine and acetyl‐CoA, and the *O*‐acetyl homoserine sulfhydrylase (MetY) synthesizes l‐homocysteine from *O*‐acetyl‐l‐homoserine and an inorganic sulfur source. In both routes, l‐homocysteine is subsequently transformed into l‐methionine by methionine synthase (MetE/MetH).

First, l‐homoserine *O*‐succinyltransferase (HST, Enzyme Commission (EC) number 2.3.1.46) catalyzes the formation of *O*‐succinyl‐l‐homoserine from l‐homoserine and succinyl‐CoA. Next, cystathionine γ‐synthase (MetB, EC 2.5.1.48) converts *O*‐succinyl‐l‐homoserine and cysteine into cystathionine. Finally, cystathionine β‐lyase (MetC; EC 4.4.1.13) cleaves cystathionine to yield l‐homocysteine. This intermediate is subsequently transformed into l‐methionine by methionine synthase (MetE/MetH). By contrast, the direct‐sulfurylation pathway is shorter, consisting of only two steps. l‐homoserine *O*‐acetyltransferase (HAT, EC 2.3.1.31) produces *O*‐acetyl‐l‐homoserine from homoserine and acetyl‐CoA, which is then converted to l‐homocysteine by *O*‐acetylhomoserine sulfhydrylase (MetY; EC 2.5.1.49) using an inorganic sulfur donor (Scheme 1). Most bacteria predominantly use the direct‐sulfurylation pathway, which uses MetAA or MetXA (Scheme 2) to activate l‐homoserine through an acylation step. However, some bacteria, including *Escherichia* *coli* (*E. coli)*, use the trans‐sulfurylation pathway.

**SCHEME 2:**
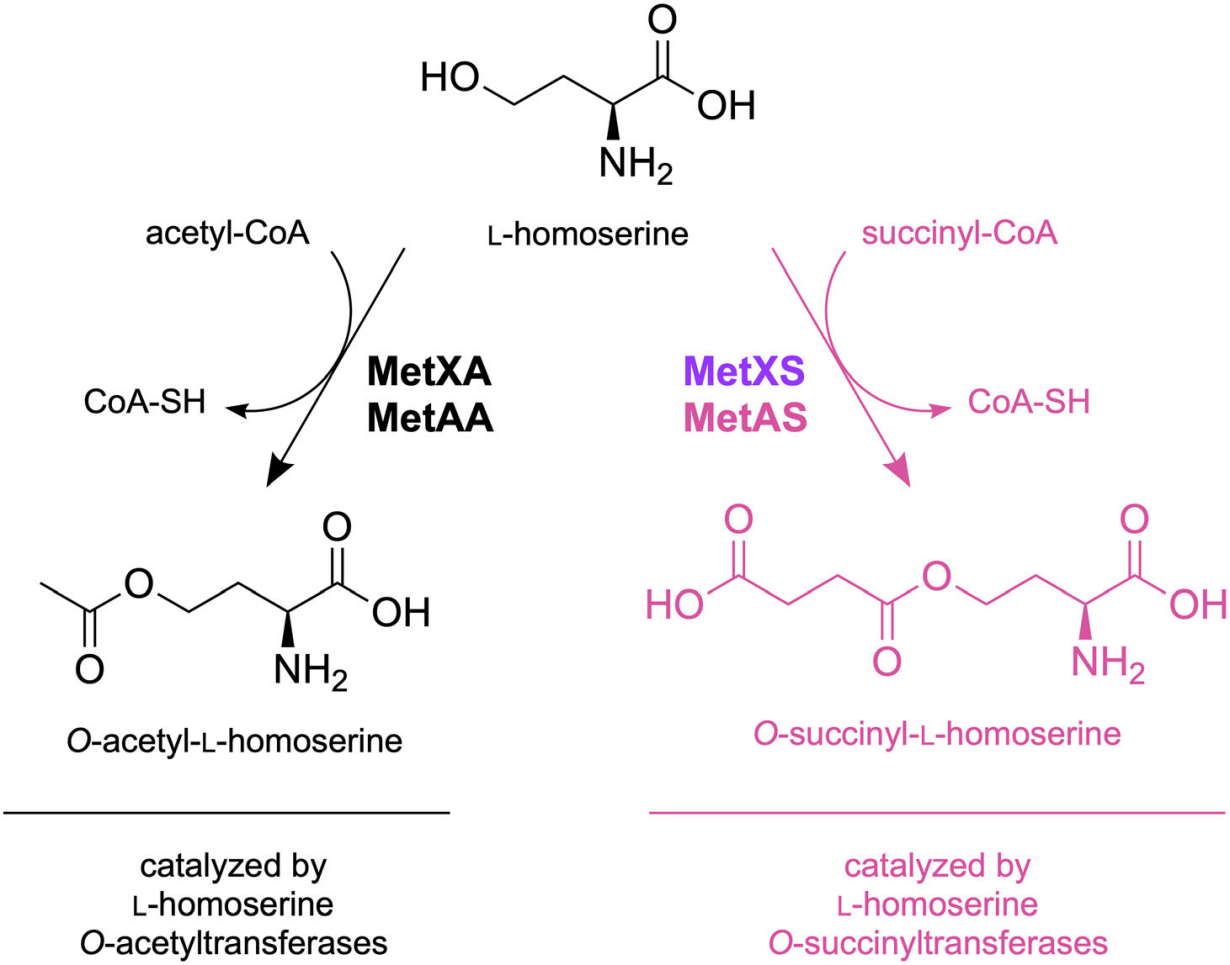
Biosynthetic roles of MetA and MetX enzymes. Enzymatic steps catalyzed in the l‐methionine biosynthetic pathways. MetXA and MetAA, l‐homoserine *O*‐acetyltransferases coded by metX and metA, respectively, and MetXS and MetAS, l‐homoserine *O*‐succinyltransferases coded by metX and metA, respectively.

Interestingly, MetA and MetX are part of phylogenetically distinct families of acyl‐l‐homoserine transferase enzymes. These two proteins do not share sequence or structural similarity, and their corresponding genes have likely evolved independently [26]. It is accepted that MetXS or MetAS acylates l‐homoserine with succinyl‐CoA to form *O*‐succinyl‐l‐homoserine, and MetXA/MetAA acylates l‐homoserine with acetyl‐CoA to form *O*‐acetyl‐l‐homoserine (Scheme 2). Furthermore, *metX*‐encoded enzymes are primarily found to catalyze transacetylations [27]. However, there are multiple exceptions of *metX*‐encoded enzymes that catalyze transsuccinylations, particularly among β‐proteobacteria [28]. In these cases, the presence of an arginine residue in the active site, which is responsible for specificity, determines the function of MetXS, making it selective for succinyl‐CoA. Due to the prevailing notion in the literature that the MetX substrate is acetyl‐CoA and the limited number of MetX‐type enzymes that have undergone biochemical characterization, these enzymes are generally annotated as HATs. A similar effect on the substrate specificity has been observed in *metA*‐encoded enzymes. While they typically function as homoserine *O*‐succinyltransferases, a single amino acid change alters their specificity, resulting in acetyltransferase activity [29].

Although the l‐methionine biosynthetic enzymes have been partially annotated from the available genome sequence of *C. necator* [30], they have not yet been functionally characterized or experimentally confirmed. Since MetX often acts as an HST despite being misannotated as an HAT, it is crucial to biochemically characterize the corresponding enzyme in *C. necator* to map the complete biosynthetic pathway in this chemolithoautotrophic bacterium.

Here, we report the identification, heterologous expression, and in vitro characterization of the HST from *C. necator* (*Cn*MetXS). Several biochemical parameters were determined, including the pH and temperature optima, thermostability, substrate scope, and kinetic parameters for succinyl‐CoA and l‐homoserine. Furthermore, we determined the X‐ray structure at 1.86 Å resolution of *Cn*MetXS, which reveals its role as a transsuccinylase in the biosynthesis of l‐methionine in *C. necator*. Alongside an analysis of the genomic context of *metXS* in *C. necator*, we present biochemical evidence indicating that this autotroph is among the few β‐proteobacteria capable of using the direct‐sulfurylation pathway for l‐methionine biosynthesis, even in the presence of the HST *Cn*MetXS. This study paves the way for metabolic engineering strategies to produce l‐methionine in *C. necator*.

## 2 Results and Discussion

### 2.1 Genomic Context of *MetXS* in *C. necator*

We first identified a candidate gene encoding the putative MetXS on chromosome 1 of *C. necator*. To further assess whether this gene indeed encodes the targeted MetXS enzyme involved in the l‐methionine biosynthesis of *C. necator*, we analyzed its genomic context. The *metXS* gene (NCBI CP196507.1) has been functionally annotated as both a putative acetyltransferase and succinyltransferase [31, 32], although these annotations were not supported by biochemical or physiological characterization. A genome‐wide homology search identified only a single copy of *m* *etXS* in the *C. necator* H16 genome.

In the immediate genomic vicinity of *metXS*, a gene encoding MetW is present. MetW has been characterized as a regulatory protein required for MetX activity in γ‐proteobacteria such as *Pseudomonas syringae* [33]. Another gene related to l‐methionine metabolism, *metK1*, encoding l‐methionine adenosyltransferase (S‐adenosyl l‐methionine synthetase), is also located near the *metXS* locus. In contrast, other genes involved in l‐methionine biosynthesis, such as *metA*, *metZ1*, *metZ2*, and *metE/metH*, are distributed at distant loci on chromosome 1.

Two homologous *O*‐succinylhomoserine sulfhydrylases were annotated in *C. necator*: *metZ1* on chromosome 1 (UniProt: Q0KC18; NCBI: CAJ92453) and *metZ2* on chromosome 2 (UniProt: Q0JZ13; NCBI: CAJ97011). However, no cystathionine‐γ‐synthase (MetB) homolog could be identified in a genome‐wide search. MetZ1 and MetZ2 are sulfhydrylases homologous to MetY (Schemes 1, 3a) and specific for *O*‐succinyl‐l‐homoserine as their substrate. This suggests that the organism likely synthesizes l‐methionine via the direct‐sulfurylation pathway, even in the presence of an HST. This makes *C. necator* one of the known exceptions to the aforementioned general rule.

**SCHEME 3:**
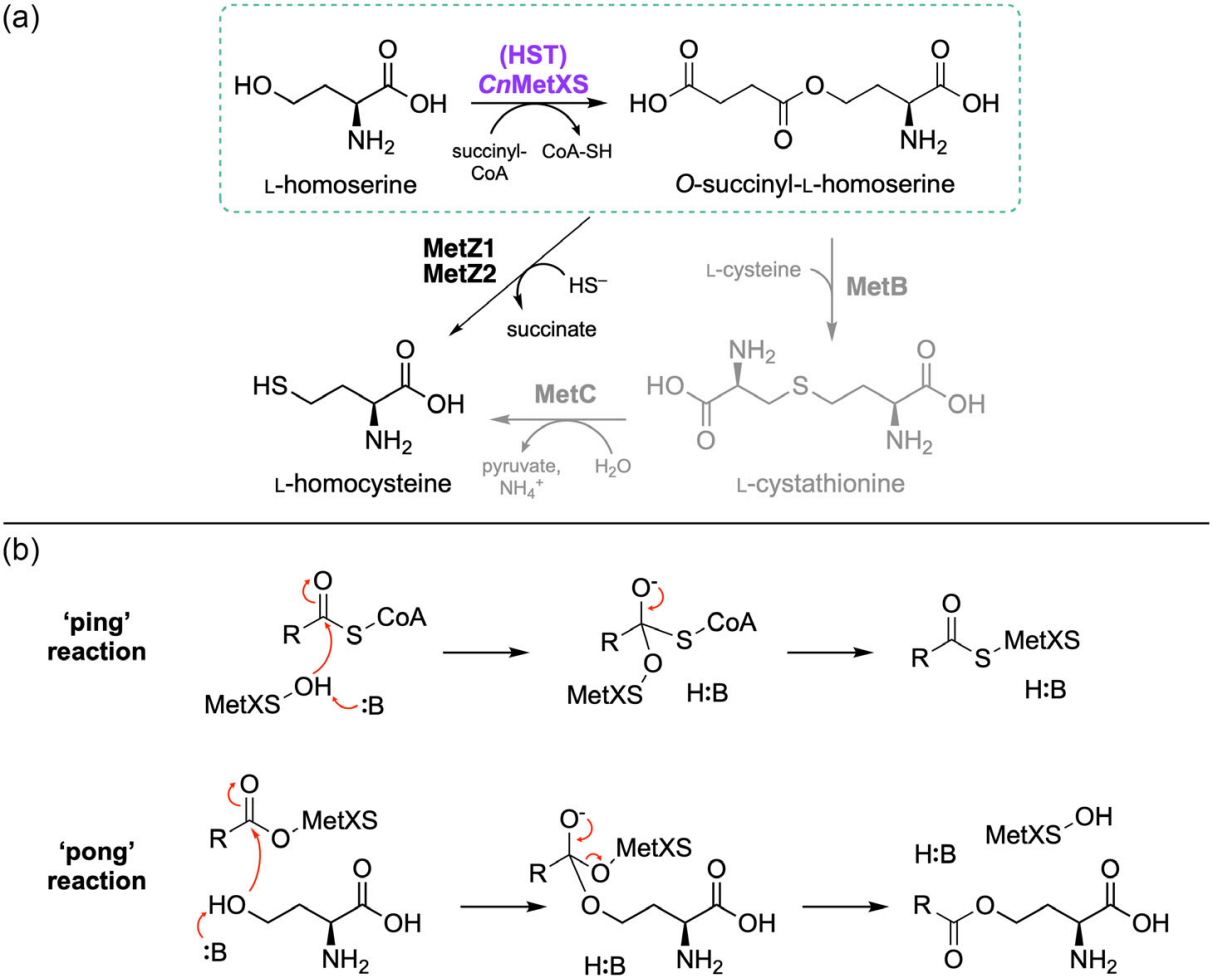
(a) *Cn*MetXS‐catalyzed *O*‐succinylation of l‐homoserine and possible sulfurylation pathways in *C. necator*: direct‐sulfurylation, catalyzed by MetZ1 and MetZ2, and transsulfurylation (gray), likely not active in *C. necator*. (b) The mechanism for the transsuccinylation suggested for the transsuccinylase in *E. coli* [35] and other transacylases, shown for *Cn*MetXS [27, 40]. The suggested mechanism for this reaction is a double replacement ping‐pong mechanism described for other homoserine *O*‐succinyltransferases.

Assimilative sulfur metabolism in *P. putida* and *P. aeruginosa* also does not follow this rule. Both harbor the *metX* gene, which encodes a succinyltransferase, and are capable of trans‐ and direct‐sulfurylation. Biochemical studies have confirmed that direct sulfurylation is strongly preferred in these organisms, while transsulfurylation is less active [34, 35]. *Corynebacterium glutamicum*, which expresses a *metA*‐encoded acetyltransferase (MetAA), can use both pathways [36], while a significant group of Gram‐positive bacteria possesses enzymes for acetyl‐dependent l‐homoserine activation coupled with transsulfurylation. This is characteristic of Firmicutes and Actinobacteria, for instance [37].

Overall, the genomic context of *metXS* in *C. necator* is consistent with a role in l‐methionine metabolism, supporting its assignment as the candidate HST and the notion that *C. necator* uses the direct‐sulfurylation pathway for l‐methionine biosynthesis (Scheme 3a).

### 2.2 Biochemical Characterization of *Cn*MetXS

After identification of the *metXS* gene in the genome of *C. necator*, the gene was subcloned into a pET‐28a(+) expression vector with an N‐terminal hexahistidine tag for purification. The recombinant enzyme was heterologously expressed in *E. coli* BL21 (DE3) and purified for subsequent biochemical characterization.

First, we investigated the catalytic properties of *Cn*MetXS to confirm its proposed role as an HST in the l‐methionine biosynthetic pathway of *C. necator*. To this end, we examined the substrate scope of the enzyme to verify its activity and assess its specificity toward potential acyl donors and acceptors. The activity assays confirmed *Cn*MetXS as a functional HST, catalyzing the conversion of succinyl‐CoA and l‐homoserine to *O*‐succinyl‐l‐homoserine (Table 1). In addition to l‐homoserine, the enzyme also accepts l‐serine as an alternative acyl acceptor. The kinetic parameters determined for *Cn*MetXS are within the range reported for other HSTs (Table 1) [27, 28, 38]. While the catalytic efficiency (*k*_cat_/*K*_m_) is approximately tenfold lower than that of the *metA*‐encoded enzyme from *E. coli*, the catalytic efficiency of *Cn*MetXS is still high and consistent with its role as a MetX‐type enzyme. These results establish *Cn*MetXS as an efficient enzyme in the biosynthesis of l‐methionine in *C. necator* and provide a suitable basis for future pathway optimization.

**TABLE 1:** Kinetic parameters for the reaction catalyzed by the l‐homoserine transsuccinylase *Cn*MetXS.

Substrate	*k*_cat_, s^–1^ a	*K*_m_, mM^a^	*k*_cat_/*K*_m_, M^–1^ s^–1^ a
Succinyl‐CoA	6.14 ± 0.46	0.31 ± 0.05	1.98 × 10^4^
Acetyl‐CoA	Inactive		
l‐homoserine	6.13 ± 0.46	1.10 ± 0.17	5.57 × 10^3^

a Reaction conditions: 20 mM KP*_i_* (with 150 mM NaCl, pH 7.5), 30 °C, substrate concentrations (0.01–0.5 mM succinyl‐CoA, 0.05–2.0 mM l‐homoserine). The second substrate was kept at saturating concentration during *K*_m_ determination for the other substrate. Initial rates were calculated from the linear range of the progress curves. Samples were derivatized with Fmoc‐Cl and analyzed by HPLC. Reactions were performed in KP*_i_* buffer due to the required sample derivatization with Fmoc‐Cl. All measurements were performed in triplicate. The determination of the kinetic parameters is described in detail in the supporting information (Section 1.4.5).

l‐Homoserine transacylases generally follow a ping‐pong double‐displacement mechanism (Scheme 3b) [38]. In the first step (the “ping” reaction), the catalytic serine attacks the acyl‐CoA donor to form a covalent acyl–enzyme intermediate. In *Cn*MetXS, Ser168 is likely deprotonated by His366 within the catalytic center, enabling transfer of the acyl group from succinyl‐CoA to the enzyme [39]. In the second step (the “pong” reaction), l‐homoserine attacks the acylated enzyme intermediate, resulting in the transfer of the acyl group to l‐homoserine and formation of *O*‐succinyl‐l‐homoserine. Given the importance of protonation states of catalytic residues and substrate functional groups, the catalytic efficiency of acyltransferases is inherently pH‐dependent. Due to that, we determined the optimum pH range of *Cn*MetXS activity under the assay conditions used in this study.

The pH range for *Cn*MetXS activity was investigated between pH 5.5 and 10.0 (Figure 1a). The highest activity was observed around neutral to slightly alkaline pH, with comparatively high initial activities at pH 7.0 and 8.0, but activity decreased at pH 7.5. This trend was consistently reproduced across independent measurements and may reflect suboptimal stabilization of charged substrates at intermediate pH.

**FIGURE 1:**
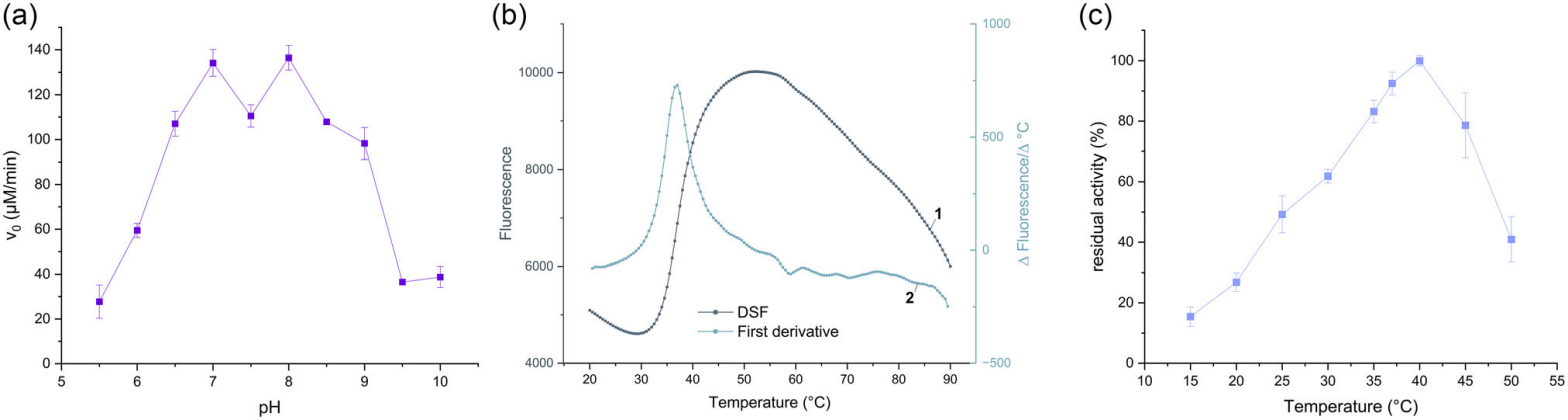
Biochemical characterization of *Cn*MetXS. (a) pH profile of *Cn*MetXS. Reaction conditions: 20 mM acetate buffer (pH 5.5), 20 mM KP_i_ buffer (pH 6.0–8.0), or 20 mM borate buffer (pH 8.5–10), all supplemented with 150 mM NaCl, 1 mM (succinyl‐CoA), 3 mM (l‐homoserine), 30 s, and 30 °C. (b) DSF assay of *Cn*MetXS. 1: Fluorescence measured in the DSF assay. 2: First derivative of the measured fluorescence. Buffer: 20 mM Tris, 150 mM NaCl, pH 7.5. (c) Temperature optimum of *Cn*MetXS. The residual activity of *Cn*MetXS is shown in %. Reaction conditions: 20 mM KP_*i*_ buffer (pH 7.5) supplemented with 150 mM NaCl, 1 mM (succinyl‐CoA), 3 mM (l‐homoserine), and 30 s. Samples were derivatized with Fmoc‐Cl and analyzed by HPLC. All measurements were performed in triplicate.

The stability of *Cn*MetXS was further examined by differential scanning fluorimetry (DSF). The melting temperature (*T*_m_) corresponds to the inflection point of the DSF signal and the maximum of the first derivative, representing the temperature at which 50% of the protein population is unfolded. Under the tested conditions, *Cn*MetXS exhibited the highest stability in 20 mM Tris buffer containing 150 mM NaCl at pH 7.5, with a melting temperature of 37.5 °C (Figure 1b). Additional stability screening of selected buffers and additives revealed that the presence of citric acid consistently decreased the thermostability of the enzyme, whereas increasing concentrations of NaCl stabilized the protein (Table S1 and Figure S3).

To further characterize *Cn*MetXS, the temperature dependence of its catalytic activity was investigated for the succinylation of l‐homoserine (Figure 1c). As the optimal growth temperature of *C. necator* is approximately 30 °C, a similar temperature optimum was expected for the enzyme. Interestingly, enzymatic activity increased up to about 40 °C; however, considering the relatively low melting temperature of 37.5 °C, temperatures above this value likely lead to partial enzyme denaturation. The relatively low melting temperature (37.5 °C) that was determined in the DSF assay indicates a limited thermostability. All kinetic measurements, including the optimal pH assay and determined Michaelis–Menten parameters, were thus performed at a temperature lower than the temperature at which the highest initial activity was observed in the thermostability assay to avoid denaturation of *Cn*MetXS.

Finally, the oligomeric state of *Cn*MetXS was analyzed by size exclusion chromatography (SEC). The enzyme predominantly eluted with an apparent molecular mass of approximately 86 kDa on a HiLoad 16/600 Superdex 75 pg column, consistent with a dimeric state in solution (ESI Figure S2, Peak 2). A minor fraction eluted at higher molecular mass (peak 1), suggesting the presence of a small population of higher‐order multimers.

### 2.3 Structure Determination of *Cn*MetXS

Historically, enzymes encoded by *metA* genes were believed to catalyze exclusively transsuccinylation reactions, whereas *metX*‐encoded enzymes were classified as HATs [27]. However, several studies have since identified exceptions to this distinction, demonstrating that some *metX*‐encoded enzymes are capable of catalyzing transsuccinylation reactions [28]. Our biochemical activity assays clearly indicate that *Cn*MetXS is a *metX*‐encoded HST, placing it among these atypical enzymes. To elucidate the molecular basis underlying this unusual substrate specificity and to gain insight into the catalytic properties of *Cn*MetXS, we determined and analyzed its crystal structure.

The crystal structure of *Cn*MetXS was solved to a resolution of 1.86 Å in space group *P*2_1_ (PDB ID 30UN, Table 2). The asymmetric unit contains two *Cn*MetXS molecules, forming a homodimer (Figure 2a). This assembly is consistent with the size‐exclusion chromatography data, indicating that *Cn*MetXS is dimeric both in solution and in the crystal. Each monomer shows a two‐domain architecture characteristic of MetX‐like homoserine acyltransferases, consisting of an α/β‐hydrolase catalytic core, comprising residues 1–195 and 303–393, and a lid domain spanning residues 196–302. (Figure 2b). The active site sits between the two domains and contains a classical α/β‐hydrolase catalytic triad formed by Ser168, Asp333, and His366 (Figure 3). The protein was soaked with 50 mM homoserine, but no density corresponding to the substrate was observed.

**TABLE 2:** Summary of data collection and refinement statistics.

	***C. necator* homoserine** ***O*‐succinyltransferase (*Cn*MetXS)**
Wavelength	1.0332 Å
Resolution range	47.2–1.86 (1.90–1.86)
Space group	P 1 21 1
Unit cell	51.51 94.392 90.025 90 91.702 90
Total reflections	502 631 (26748)
Unique reflections	71 746 (4352)
Multiplicity	7.0 (6.1)
Completeness (%)	99.3 (97.8)
Mean I/sigma(I)	8.5 (0.6)
Wilson B‐factor	25.35
R‐merge	0.146 (2.868)
R‐meas	0.172 (3.503)
R‐pim	0.090 (1.924)
CC1/2	0.996 (0.339)
Reflections used in refinement	71 487 (2733)
Reflections used for R‐free	3538 (148)
R‐work	0.1891
R‐free	0.2231
Number of non‐hydrogen atoms:	6492
Macromolecules	5903
Ligands	80
Solvent	509
Protein residues	756
RMS(bonds)	0.006
RMS(angles)	0.96
Ramachandran favored (%)	98.14
Ramachandran allowed (%)	1.86
Ramachandran outliers (%)	0.00
Rotamer outliers (%)	0.17
Clash score	3.67
Average B‐factor:	36.98
Macromolecules	36.57
Ligands	42.71
Solvent	40.78

**FIGURE 2:**
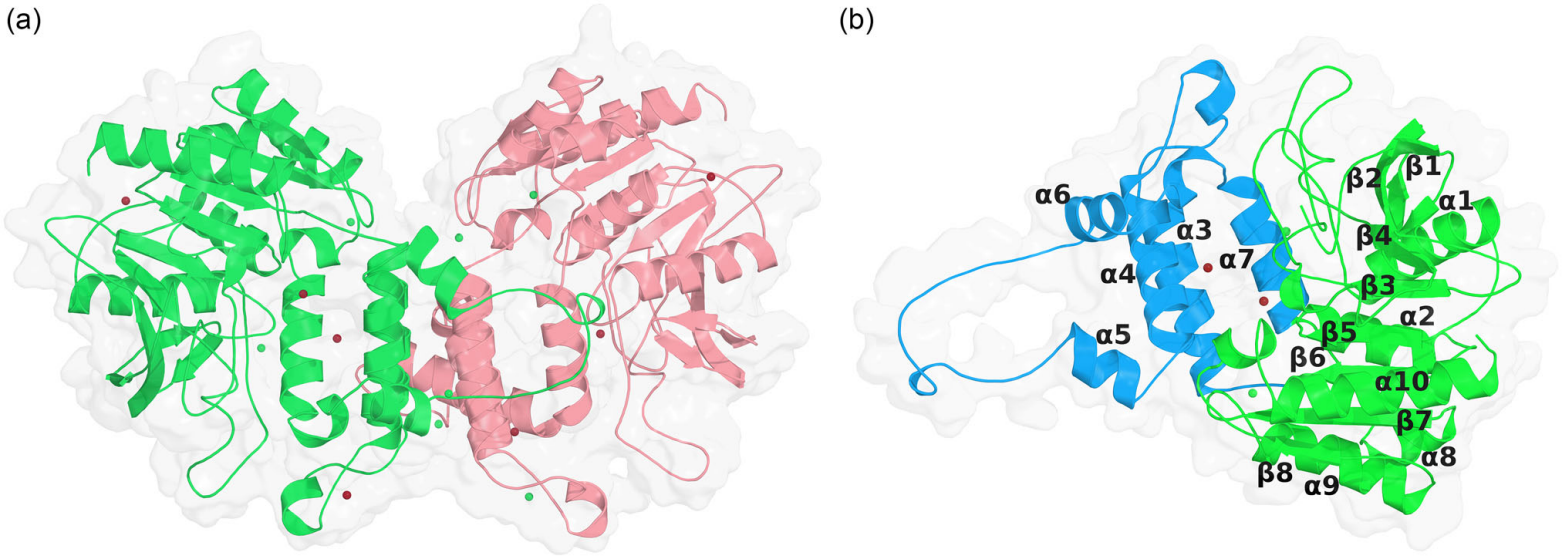
Overview of the overall structure and active site of *Cn*MetXS. (a) Overview of the *Cn*MetXS homodimer in cartoon representation. Chain A is shown in green and chain B in salmon. A transparent molecular surface is shown in gray, and ordered bromide (brown) and chloride (green) ions are shown as spheres. (b) Overview of the *Cn*MetXS chain A monomer with highlighted domains. The catalytic core is shown in green (res 1–195 and 303–393), and the lid domain in blue (res 196–302). Secondary‐structure elements are labeled.

**FIGURE 3:**
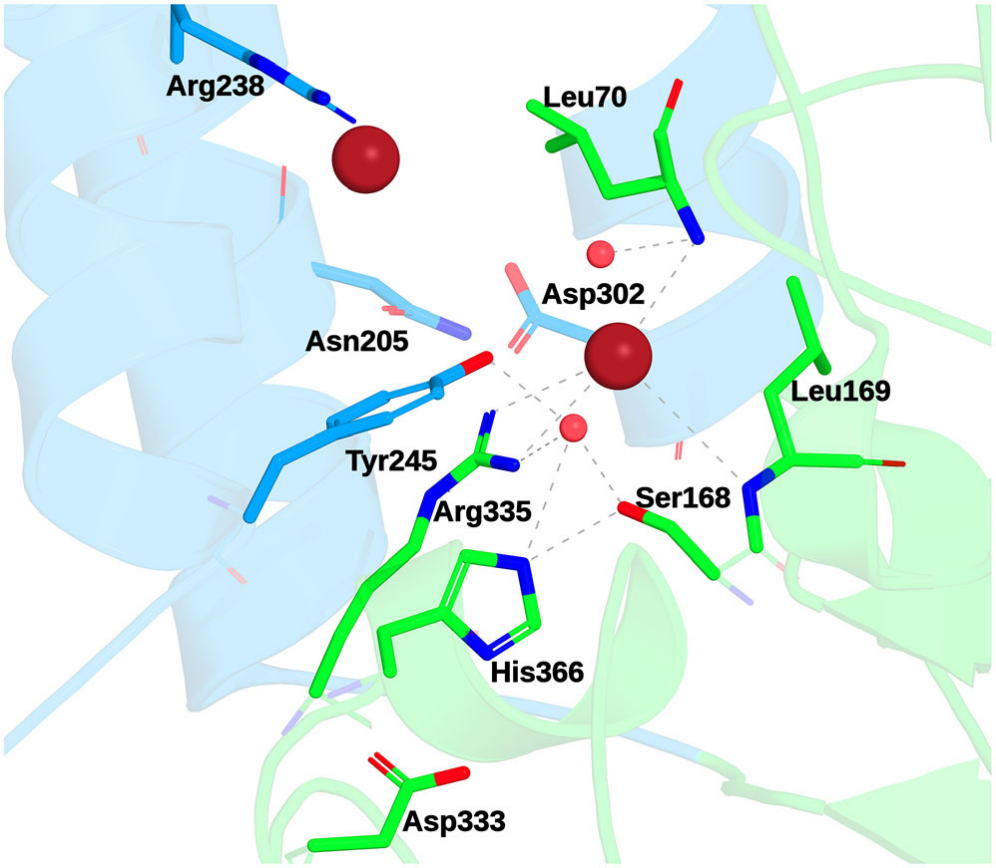
Close‐up of the active site of *Cn*MetXS chain A with key residues shown as sticks. The catalytic core and lid domain are colored as in Figure 2b. Ordered bromide ions (brown) and water molecules (red) are shown as spheres, and selected polar contacts are shown as gray dashed lines.

The active‐site composition supports classification of *Cn*MetXS as a MetX‐G2 homoserine *O*‐succinyltransferase (Figure S11).

Bastard *et al.* showed that MetX enzymes can be divided into active‐site groups with differing acyl‐CoA specificities, with MetX‐G1 enzymes corresponding to HATs and MetX‐G2/G3 enzymes to HSTs [28]. The succinyl‐CoA specificity in MetX‐G2 and G3 is associated with a key arginine at logo positions 17 and 11, respectively, which is proposed to stabilize the carboxylate of the succinylated serine intermediate [28]. Mutation of this arginine to leucine removed succinyl‐CoA activity and shifted activity toward acetyl‐CoA, whereas the opposite leucine‐to‐arginine substitution in a MetX‐G1 enzyme induced succinyltransferase activity [28]. In *Cn*MetXS, this residue corresponds to Arg335 (Figure S11). The further classification of *Cn*MetXS into G2 over G3 is the presence of Leu169 adjacent to the catalytic Ser168.

Enzymes belonging to the MetX‐G2 group typically contain a leucine residue adjacent to the catalytic serine, whereas in MetX‐G3 enzymes this position is most often occupied by methionine, although tyrosine residues have also been observed. *Cn*MetXS also conserves the predicted oxyanion‐hole residues Leu70 and Leu169 and the conserved homoserine‐binding residues Arg238 and Asp367 (Figures 2a and S11).

Structural comparison with representative MetX homologs further demonstrates the conservation of the *Cn*MetXS architecture. Superposition of *Cn*MetXS chain A with MtHAT (8F2L) [41], MsHAT (6IOG) [42], LiHAT (2PL5) [43], and HiHAT (2B61) [44] yielded Cα RMSDs of 0.9–1.7 Å over 288–302 aligned Cα atoms (Fig S12 and Table S2). Consistent with this overall structural conservation, the catalytic Ser168‐Asp333‐His366 triad and residues lining the substrate‐binding site occupy closely corresponding positions in the superimposed structures (Fig. S12d). Asn71 occupies the position of a conserved polar Thr/Ser residue in the homologs, preserving a polar side chain at this site while positioning its terminal group slightly farther toward the periphery of the substrate‐binding pocket. In contrast, Arg335, associated with succinyl‐CoA specificity in *Cn*MetXS, occupies the structural position corresponding to a leucine residue in all four HAT homologs (Fig S12c). Thus, *Cn*MetXS retains the conserved MetX scaffold and active‐site organization while differing locally at positions associated with acyl‐donor specificity.

Several halide ions were modeled in the structure using both electron‐density and anomalous‐scattering evidence. Large peaks were observed in the 2Fo–Fc map, but unambiguous assignment of all peaks was challenging. Therefore, anomalous difference density was used as an additional tool for ion identification. Peaks with anomalous signals greater than 5σ were modeled as bromide, whereas strong 2Fo–Fc peaks with absent or weaker anomalous signals were modeled as chloride. One bromide ion in chain A is located near Ser168, in the oxyanion hole, formed by the backbone amides of Leu70 and Leu169, and is making electrostatic interactions with Arg335 (Figure 3).

This bromide might approximate the position of where the carbonyl oxygen of the succinylated serine intermediate would bind. The abundance of ordered halide ions in the structure may also be consistent with the thermal stabilization observed at increasing NaCl concentrations in the DSF assay, suggesting that ionic strength or specific anion binding contributes to stabilization of *Cn*MetXS.

The interaction between the monomers is predominantly mediated through the lid domains. In particular, helices α3 and α4 of the lid domain closely resemble the corresponding helices in MetX transferases [42, 43] and form the core of the intersubunit contacts in *Cn*MetXS (Figure 2a,b). Sagong et al. previously observed that although most of the lid domain is conserved among the MetX‐HAT family enzymes, the α5–α6 helices and connecting loop vary substantially between species, producing different interactions between the monomers and potentially contributing to species‐specific differences in dimer organization [42]. The corresponding region in *Cn*MetXS, Thr244–Ala291, is also distinct from the other structurally similar MetX‐HATs (Figure S12).

More notably, the two *Cn*MetXS monomers adopt distinct conformations of this region (Figures 4a,b, S8, and S9).

**FIGURE 4:**
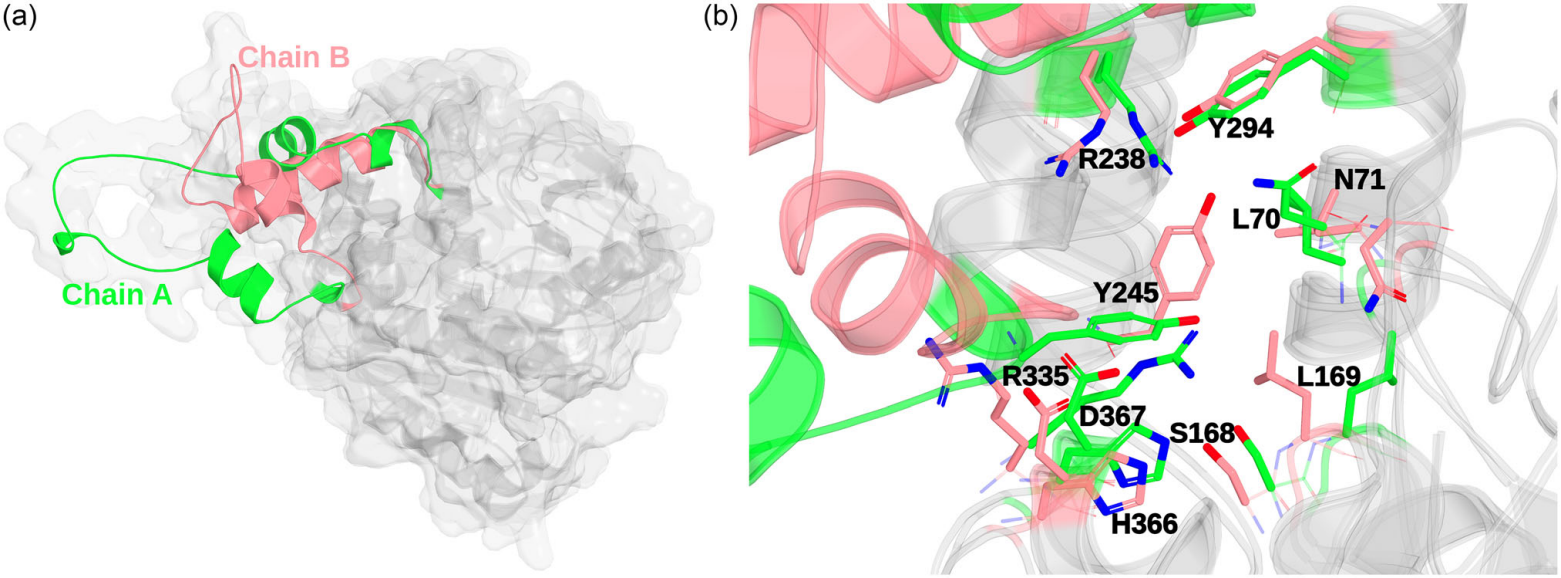
Structural comparison of the *Cn*MetXS monomers. (a) Conformational differences between chains A and B in the α5–α6 lid region. Chains A and B were superimposed and are shown as cartoon diagrams, with the overall structures and molecular surface shown in transparent gray. The α5–α6 lid region (Thr244–Ala291) is highlighted in green for chain A and salmon for chain B. (b) Close‐up of the superimposed active sites. Key active site residues are shown as sticks and colored by chain, green for chain A and salmon for chain B.

Superposition of chains A and B reveals a divergence starting at Gly241, near the end of helix α4, leading to a large shift of the Thr244–Ala291 region, seemingly adopting two distinct conformations (Figure 4a). This shift generates an asymmetric active site architecture. In chain B, the loop containing Tyr245 is rearranged into the active site, seemingly narrowing the entrance (Figure 4b). In addition, Arg335 is positioned near the catalytic center in chain A but is displaced away from the Ser168 region in chain B. Thus, chain A appears more compatible with productive acyl‐donor binding, whereas chain B adopts a rearranged state that is less favorable for catalysis. However, it cannot be ruled out that the chain B conformation is influenced by crystal packing, as this lid segment forms contacts with symmetry‐related molecules. PISA analysis [45] identified a buried surface area of 380 Å^2^ and an estimated Δ*G* of −3.6 kcal per mol for this crystal contact, indicating that the interaction is sufficiently substantial to stabilize the observed conformation. In addition, the observed electron density around this lid segment in chain A is weaker, and the B factors are higher, indicating that there is some inherent flexibility to this region. Therefore, the closed/rearranged state of chain B should be interpreted carefully and may not necessarily represent the predominant dimer conformation in solution.

To obtain more detailed information on substrate binding modes, soaking and cocrystallization experiments were performed with succinyl‐CoA and l‐homoserine. However, no interpretable electron density was observed for either substrate or for a succinylated Ser168 intermediate. One possible explanation is that the high halide concentrations present in the crystallization condition, and the subsequent halide ions observed in and around the active site, interfere with substrate binding. Therefore, to gain further insight into substrate recognition, in silico docking experiments were performed.

To investigate how l‐homoserine could be accommodated after formation of the acyl‐enzyme intermediate, Ser168 was modeled in silico as succinylated, and l‐homoserine was docked into the chain A active site (Figure 5). Using our crystal structure and YASARA, a docking pose was obtained in which l‐homoserine is stabilized in the active site by several key residues, including Asn71, succinylated Ser168, Tyr245, Tyr294, Arg335, and His366 (Figure 5). The hydroxyl group of l‐homoserine is positioned in close proximity to the succinyl group, consistent with a geometry that would allow for a nucleophilic backside attack. Importantly, Arg335, the arginine corresponding to the previously described MetX‐G2 succinyl‐specificity arginine, interacts with the terminal carboxylate of the modeled succinyl group. This supports its proposed role in stabilizing the succinylated intermediate in *Cn*MetXS (Figure 5).

**FIGURE 5:**
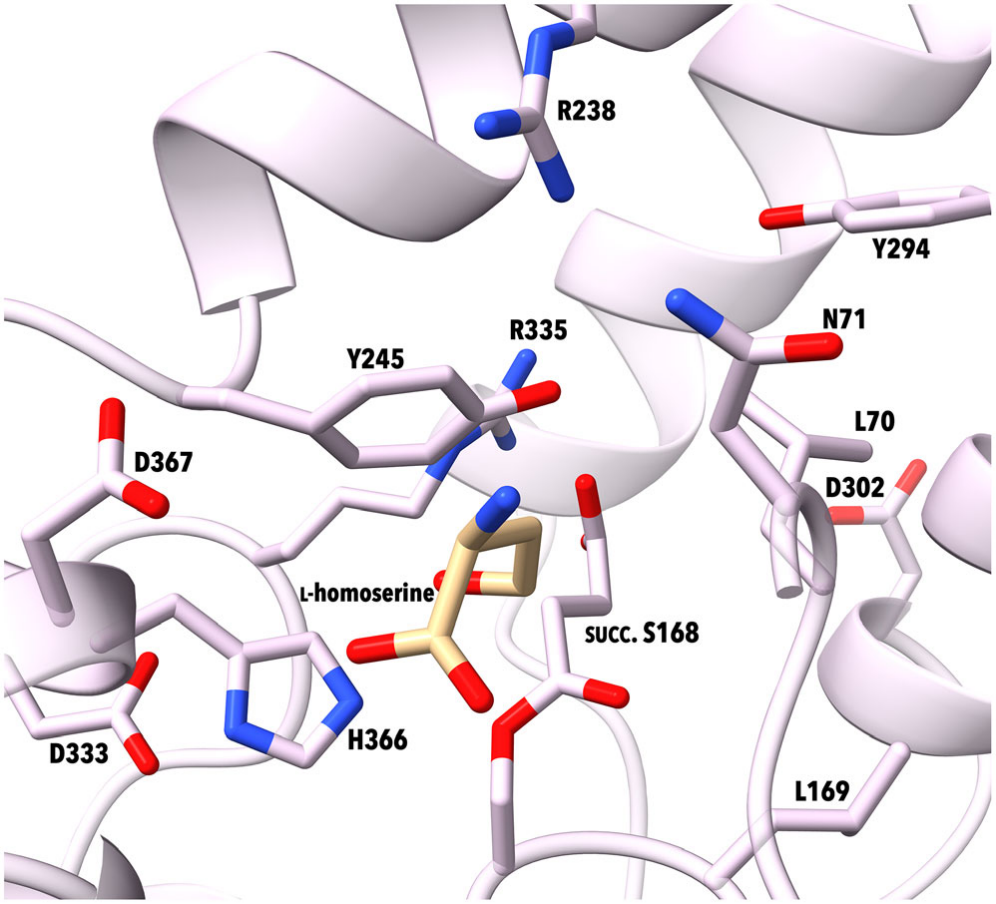
Docking result of l‐homoserine to 168‐succinyl *Cn*MetXS, chain A. Highly conserved residues in MetXS‐G2 enzymes are displayed (light pink sticks). succ. Ser168: succinylated serine in position 168. The docking parameters are described in the supporting information (section 1.5).

We identified a docking pose in which Arg335 interacts with the succinyl group of the succinylated Ser168, providing a structural explanation for the observed substrate specificity.

The docking experiments revealed that the differently shaped active sites of the two monomers might influence catalytic activity. While l‐homoserine could be successfully docked into the active site of chain A of succinylated *Cn*MetXS, no reasonable docking position was obtained for chain B. The different docking results can be attributed to the distinct conformations of the active site. In chain A, the active site is accessible, while in chain B it is narrowed by a tyrosine residue (Tyr245) whose position is influenced by the rearranged section mentioned above (Thr244–Ala291, Figures 4b and S10). Furthermore, the important Arg335 is moved out of the active site in chain B. Consequently, the reduced accessibility of the active site in chain B, along with conformational changes of other residues in the active site, likely limits ligand accommodation, thereby rationalizing the observed differences in the docking results. These structural observations are consistent with the biochemical characterization of *Cn*MetXS, which demonstrated a clear preference for succinyl‐CoA as the acyl donor (Scheme 3a).

## 3 Conclusion

In this study, we identified the *metXS* gene from *C. necator* H16 as encoding a homoserine *O*‐succinyltransferase involved in l‐methionine biosynthesis. *Cn*MetXS was biochemically characterized, and substrate scope and kinetic analyses confirmed that the enzyme catalyzes the succinylation of l‐homoserine using succinyl‐CoA as the preferred acyl donor, while only limited activity was observed with alternative substrates. Although the catalytic efficiency of *Cn*MetXS is lower than that reported for the *metA*‐encoded enzyme from *E. coli*, the observed catalytic efficiency is high and consistent with its physiological role in l‐methionine biosynthesis in *C. necator*. Structural analysis further provided insight into the molecular basis of substrate recognition and catalysis. The crystal structure revealed asymmetric conformations of the active‐site entrance, with chain A adopting an arrangement more compatible with productive ligand accommodation than chain B. This asymmetry seems to start from a rearrangement of the adjacent lid region that propagates into the active site. However, given the structural variability of this region among MetX homologs and the substantial crystal contact associated with the chain B conformation, the functional significance of the crystallographic asymmetry in solution remains unresolved. In silico docking experiments support the experimentally observed substrate specificity and indicate favorable positioning of l‐homoserine within the active site of the succinylated enzyme intermediate. Sequence and structural features place *Cn*MetXS within the MetX‐G2 group of HSTs, highlighting the diversity of catalytic functions among *metX*‐encoded enzymes that have evolved convergently with *metA*‐encoded transsuccinylases.

The results of the biochemical characterization and genomic analysis indicate that *C. necator* most likely follows the direct sulfurylation pathway even though the presence of succinyltransferases is usually linked with the transsulfurylation pathway.

Together, these findings expand our understanding of l‐methionine biosynthesis in β‐proteobacteria and provide the first detailed characterization of a MetX‐type HST from *C. necator*. Beyond its fundamental biochemical relevance, this work establishes MetXS as a target for future metabolic engineering efforts aimed at improving l‐methionine biosynthesis in *C. necator*, an increasingly attractive chassis for sustainable bioproduction.

## Funding

This study was supported by H2020 Marie Skłodowska‐Curie Actions (955740) and Nederlandse Organisatie voor Wetenschappelijk Onderzoek (VI.Vidi.213.025).

## Conflicts of Interest

The authors declare no conflicts of interest.

## Supporting information

Supplementary Material
