# Supplementary material for: Characterization and Crystal Structure Elucidation of the l‐Homoserine O‐Succinyltransferase MetXS From Cupriavidus necator H16
Source: Chembiochem. 2026 Oct 20;27(20):e70564. doi: 10.1002/cbic.70564 (PMC13683966; doi:10.1002/cbic.70564)
Supplement: Supplementary file 1 [file CBIC-27-e70564-s001.pdf]

SUPPLEMENTARY INFORMATION FOR

Characterization and Crystal Structure Elucidation of the L-Homoserine *O*-Succinyltransferase MetXS from *Cupriavidus necator* H16

Hannah Pia Franziska Meier,[a] Rick Oerlemans,[a] Henrik Terholsen,[a] Matthew R. Groves,[a] Lars Lauterbach[b] and Sandy Schmidt*[a]

[a] H.P.F. Meier, Dr. R. Oerlemans, Dr. H. Terholsen, Prof. Dr. M. R. Groves, Prof. Dr. S. Schmidt
Department of Chemical and Pharmaceutical Biology
Groningen Research Institute of Pharmacy, University of Groningen
Antonius Deusinglaan 1, 9713 AV Groningen (The Netherlands)
E-mail: s.schmidt@rug.nl

[b] Prof. Dr. L. Lauterbach
iAMB - Institute of Applied Microbiology
RWTH Aachen University
Worringer Weg 1, 52074 Aachen (Germany)

Table of Contents

1 EXPERIMENTAL SECTION 3

1.1 General procedures 3

1.2 Expression, purification and analysis of CnMetXS 3

1.3 Purification of the CnMetXS dimer by Size-Exclusion Chromatography 3

1.4 In vitro biochemical assays and product analysis 3
1.4.1 General enzyme assay 3
1.4.2 Optimal pH assay 4
1.4.3 Differential scanning fluorimetry (DSF) 4
1.4.4 Thermostability assay 4
1.4.5 Kinetics 4
1.4.6 Sample preparation and derivatization 4
1.4.7 HPLC analysis 4

1.5 Crystallization and structure determination 5

1.6 In silico docking 5

2 SEQUENCES 5

3 SUPPLEMENTARY FIGURES 6

3.1 Biochemical characterization of CnMetXs 6

3.2 Structural analysis of CnMetXS 9
3.2.1 Differences between the chain A and B in the crystal structure of CnMetXS 10
3.2.2 Characterization of CnMetXS as a member of the MetX-G2 group 10
3.2.3 Structure of the conserved lid-domain and comparison to other structurally similar MetX-HATs 11
11

3. SUPPLEMENTARY TABLES 12

4. REFERENCES 14

1 Experimental section

1.1 General procedures

Commercially available reagents were used without further purification. (2*S*)-2-amino-4-hydroxybutanoic acid (L-homoserine) was purchased from Enamine Ltd. (Kyiv, Ukraine) and used as received. Succinyl-coenzyme A sodium salt was purchased from Sigma-Aldrich (Darmstadt, Germany) and used as received. Acetyl-coenzyme A trilithium salt was purchased from VWR B.V. (Amsterdam, The Netherlands) and used as received. All CoA substrates were dissolved to a final concentration of 50 mM (succinyl-CoA) and 25 mM (acetyl CoA) in 50 mM KPi buffer, pH 7.0 and immediately stored in 20 µL aliquots at -80 °C until further use. L-homoserine was diluted to a final concentration of 10 mM in Milli-Q water and stored in 250 µL aliquots at -20 °C. All biotransformations were carried out under non-inert conditions. High-performance liquid chromatography analysis was performed on a Shimadzu LC-10AT equipped with a photodiode array detector. Columns and conditions for HPLC analyses are indicated separately. L-Valine was used as an internal standard.

1.2 Expression, purification and analysis of *Cn*MetXS

The gene encoding for MetXS from *Cupriavidus necator* H16 (*Cn*MetXS) was ordered subcloned using the XhoI and NcoI restriction sites into a pET-28a(+) expression vector from Twist Bioscience (San Francisco, USA). *E. coli* BL21 (DE3) (Invitrogen) was used for expression of *Cn*MetXS. Expression and purification of N-terminally His-tagged *Cn*MetXS was performed by first preparing a preculture in 20 mL *lysogeny broth* (LB, 10 g/L tryptone, 10 g/L NaCl, 5 g/L yeast extract) supplemented with 50 µg/mL kanamycin in 100 mL baffled Erlenmeyer flasks. From the overnight culture, 500 mL LB medium was inoculated into a 3 L baffled flask to an initial cell density of OD_{600} 0.1 and incubated at 37 °C, 120 rpm. Gene expression was induced at a cell density of ~0.6 by adding 100 µM isopropyl β-D-1-thiogalactopyranoside (IPTG). After the cells were grown at 20 °C for 20 hours, the cells were harvested and resuspended in lysis buffer (20 mM Tris-HCl, 150 mM NaCl, 30 mM imidazole, pH 7,5, 10% glycerol) and disrupted by sonication (70% duty cycle, power control of 7, 6 cycles of 30 s ON/30 s OFF). The soluble fraction of the cell lysate was recovered after centrifugation (13,000 × *g* for 90 min). Nickel-nitrilotriacetic acid (Ni-NTA) resin (GE Healthcare, USA) was used to isolate His-tagged protein. Cell free extracts (CFEs) were incubated with 3 mL (1 CV) Ni-sepharose for 1 hour at 4 °C. They were then washed using 5 CVs washing buffer (20 mM Tris-HCl, 150 mM NaCl, 30 mM imidazole, pH 7,5, 10% glycerol). Elution was performed by adding three times 2 CVs elution buffer (20 mM Tris-HCl, 150 mM NaCl, 300 mM imidazole, pH 7,5, 10% glycerol). Purified protein-containing fractions were confirmed by SDS-PAGE and then desalted using a PD-10 column according to the manufacturer's instruction (Cytiva, USA) with storage buffer (20 mM TRIS buffer supplemented with 150 mM NaCl, pH 7.5, 10% glycerol). The desalted protein was concentrated to about 28 mg/mL, snap frozen in liquid nitrogen and stored at −80 °C for further use. Protein concentration was determined by the Bradford protein assay (Bio-Rad) using bovine serum albumin (BSA) to generate a standard curve.

1.3 Purification of the *Cn*MetXS dimer by Size-Exclusion Chromatography

The oligomeric state of *Cn*MetXS was analyzed and the dimeric fraction isolated by size-exclusion chromatography (SEC). SEC was performed using a HiLoad™ 16/600 Superdex 75 pg column (GE Healthcare) connected to an ÄKTA™ purification system. Prior to sample injection, the column was equilibrated with SEC buffer consisting of 20 mM Tris-HCl, 150 mM NaCl, pH 7.5. All chromatographic runs were carried out at room temperature. Purified and concentrated *Cn*MetXS obtained from the preceding purification step was loaded onto the column using a sample volume appropriate for the column capacity. Elution was performed isocratically with SEC buffer at a constant flow rate, and the absorbance at 280 nm was continuously monitored to detect protein-containing fractions. Protein eluted predominantly as a major peak corresponding to an apparent molecular mass of approximately 86 kDa, consistent with the dimeric form of *Cn*MetXS. A minor earlier-eluting peak corresponding to higher-order oligomeric species was also observed. Fractions corresponding to the dimeric peak were collected and analyzed by SDS–PAGE to confirm purity. Fractions containing the dimeric CnMetXS were pooled and concentrated to approximately 28 mg/mL. The purified protein was aliquoted, flash-frozen, and stored at −80 °C until further use. The isolated dimeric fraction was used for subsequent enzyme activity assays and protein crystallization experiments.

1.4 In vitro biochemical assays and product analysis

1.4.1 General enzyme assay

The enzyme assays were performed in a total reaction volume of 25 µL. For each reaction, the required buffer volume was calculated based on the volumes of enzyme and substrates according to the following equation:

$$V_{buffer} = \left(25 - V_{enzyme} - V_{acyl\,donor} - V_{L-homoserine}\right)\,\mu L$$

All solutions used in the enzyme assays were kept on ice throughout the experimental procedure unless stated otherwise. Prior to starting the reactions, an Eppendorf ThermoMixer® was set to 600 rpm and allowed to equilibrate to the desired reaction temperature. The enzyme stock solution (20 mg/mL) was diluted to a concentration approximately tenfold higher than the final concentration used in the reaction mixture. The volume corresponding to this enzyme dilution was considered in the buffer

volume calculation. Stock solutions of L-homoserine (50 mM) and the respective acyl donor (succinyl-CoA or acetyl-CoA) were similarly diluted to concentrations approximately tenfold higher than their intended final concentrations in the assay.

The calculated volume of buffer was pipetted into a round-bottom 96-well plate, which was kept on ice during preparation. Subsequently, the required volumes of L-homoserine and the acyl donor were added to each well. The plate was then placed in the ThermoMixer®, and the reaction was initiated by addition of the enzyme. After enzyme addition, the plate was sealed with a lid and incubated under agitation at 600 rpm.

The enzymatic reactions were quenched by adding 25 µL of HPLC-grade acetonitrile. Immediately after quenching, the plate was returned to ice. Precipitated protein was subsequently removed by centrifugation at 4000 × *g* for 30 min.

1.4.2 Optimal pH assay

The effect of pH on *Cn*MetXS activity was evaluated by measuring enzyme activity across a defined pH range. Reactions (25 µL) contained 200 µM *Cn*MetXS, 3 mM L-homoserine and 1 mM succinyl-CoA in 20 mM acetate buffer (pH 5.5), 20 mM KPi buffer (pH 6.0–8.0), or 20 mM borate buffer (pH 8.5–10), all supplemented with 150 mM NaCl. Reactions were performed at 30 °C for 30 seconds, and product formation was monitored by HPLC (UV detector, 265 nm) after sample derivatization (see section 1.4.6). Control reactions without enzyme were included. All experiments were performed in triplicates.

1.4.3 Differential scanning fluorimetry (DSF)

Differential scanning fluorimetry (DSF) was used to evaluate the thermal stability of *Cn*MetXS under a range of buffer conditions using a ThermoFluor-inspired 96-well buffer screen, with the individual conditions listed in Table S1. A concentrated *Cn*MetXS–dye mixture was prepared containing 50 µM CnMetXS and 25× SYPRO Orange dye. 5 µL of the mixture was added to each well of a 96-well PCR plate, followed by addition of 45 µL of buffer-screen solution to the corresponding wells. The final assay volume was 50 µL, containing 5 µM *Cn*MetXS and 2.5× SYPRO Orange dye in each well. The plate was sealed with transparent film and incubated at room temperature for 15 min. Prior to measurement, the plate was centrifuged at 1000 × *g* for 1 min to remove air bubbles. Thermal unfolding was monitored using a BioRad CFX96 Real-Time PCR system. Samples were equilibrated at 20 °C for 2 min and subsequently heated from 20 to 90 °C in 0.5 °C increments, with a 30s hold at each temperature before fluorescence measurement with the HEX channel. Melting temperatures (T_m) were determined from the resulting fluorescence–temperature curves by analysis of the first derivative of the fluorescence signal to find the inflection points.

1.4.4 Thermostability assay

The effect of temperature on *Cn*MetXS activity was evaluated by measuring enzyme activity across a defined temperature range. Reactions (25 µL) contained 200 µM *Cn*MetXS, 3 mM L-homoserine and 1 mM succinyl-CoA in 20 mM KPi buffer (pH 7.5), supplemented with 150 mM NaCl. Reactions were performed at 30 °C for 30 seconds, and product formation was monitored by HPLC (UV detector, 265 nm) after sample derivatization (see section 1.4.6). Control reactions without enzyme were included. All experiments were performed in triplicates.

1.4.5 Kinetics

Steady-state kinetic parameters of *Cn*MetXS were determined by measuring initial reaction rates at varying substrate concentrations (0.01–0,5 mM succinyl-CoA, 0,05–2,0 mM L-homoserine). CoA substrates were dissolved to a final concentration of 50 mM (succinyl-CoA) and 25 mM (acetyl CoA) in 50 mM KPi buffer, pH 7.0 and immediately stored in 20 µL aliquots at -80 °C until further use. L-homoserine was diluted to a final concentration of 10 mM in Milli-Q water and stored in 250 µL aliquots at -20 °C. Initial rates were calculated from the linear range of the progress curves. Kinetic parameters were calculated using OriginPro2020 (OriginLab Corporation, Northampton, MA, USA) using a nonlinear regression fit to the Michaelis–Menten equation. All measurements were performed in triplicates.

1.4.6 Sample preparation and derivatization

Product formation was analyzed by HPLC. For this, the reaction mixtures were quenched by adding HPLC-grade acetonitrile to a final volume of 100 µL. The precipitated protein was removed by centrifugation in the plate at 4000 x *g* for 30 minutes. 75 µL of the supernatant were transferred to a new round-bottom 96-well plate. The internal standard L-valine was added to a final concentration of 0.25 mM. For derivatization, 16 µL of borate buffer (200 mM, pH 8.0) was added to each sample, followed by 10 µL of Fmoc-Cl solution (20 mM in acetonitrile). The samples were incubated for 10 min at room temperature to allow complete derivatization.

1.4.7 HPLC analysis

Following derivatization, the samples were placed directly in the HPLC autosampler and injected without further preparation. Chromatographic separation was performed using a Phenomenex Kinetex® EVO C18 column (5 µm, 150 × 4.6 mm, 100 Å) with a total run time of 19 min, applying a linear gradient from 5% to 95% ACN supplemented with 0,1 % trifluoroacetic acid (TFA) in water supplemented with 0,1 % TFA. The column temperature was kept at 40 °C. Calibration curves were recorded using serial dilutions. Data acquisition and analysis were performed using the instrument's standard software.

1.5 Crystallization and structure determination

*Cn*MetXS was subjected to sitting-drop vapour-diffusion crystallization screening using commercially available sparse-matrix screening kits (PACT premier, MORPHEUS and JCSG plus, Molecular Dimensions). Drops composed of 0.5 µL protein solution (28 mg/mL) and 0.5 µL reservoir solution were set and equilibrated against 30 µL of reservoir buffer at 18 °C. Initial crystals were observed after 1 - 2 days in conditions A2, B2, D2, G2, and H2 of MORPHEUS. Condition B2 (0.1 M MES/Imidazole pH 6.5, 10% w/v PEG 8000, 20% v/v ethylene glycol, 30 mM sodium fluoride, 30 mM sodium bromide and 30 mM sodium iodide) was selected for optimization, as it yielded the largest crystals. Optimization was performed by varying the pH (6.0 - 7.0) of the buffer mix and the concentration of the precipitant mix (20 - 40%) whilst keeping the halogen additives fixed. The optimized condition yielding the best-diffracting crystals consisted of 0.1 M MES/Imidazole pH 7, 12% w/v PEG 8000, 24% v/v ethylene glycol, 30 mM sodium fluoride, 30 mM sodium bromide and 30 mM sodium iodide.

Crystals were harvested directly from the crystallization drops and flash-cooled in liquid nitrogen. X-ray diffraction data were collected at beamline P11 of the Deutsches Elektronen-Synchrotron (DESY, Hamburg, Germany) at 100 K. Data were automatically processed at the beamline with XDS[1] and scaled with AIMLESS.[2] The structure was solved by molecular replacement with Phaser[3] using the AlphaFold[4–6] model AF-Q0KF58-F1-model_v4 as the search model. The structure was then further refined by iterative cycles of manual model building in Coot[7] and refinement with phenix refine.[8]

Data collection and refinement statistics are reported in Table S2. The final structure was deposited in the protein data bank (PDB) with accession code 30UN.

1.6 In silico docking

Molecular docking experiments were performed using the VINA docking algorithm in the YASARA structure software package (version 24.4.10, Vienna, Austria). Docking was performed in a simulation cell measuring 15 Å around the catalytic Ser168 residue. The residues Tyr245, Arg335, His366, Phe369, Leu370 and the succinylated Ser168 of Chain A were kept flexible, while all other residues were rigidified. One hundred docking runs were performed. Similar docking poses (with a root mean square deviation of less than 3.0 Å) were clustered together. UCSF Chimera (San Francisco, CA, USA) was used for visualization.

2 Sequences

***Cn*MetXS**

Uniprot ID: A0A367PPA7

NCBI accession number: CP196507.1

- Nucleotide sequence (with N-terminal His-tag):

ATGCATCATCACCATCACCACACTGATGTCGCCCTGCCGACCGCCGCGCCTGCCGCGTTCGACCTGCCGCCCGATTCG
GTCGGCGTGGTTGCGCCGCAGCGCATGCACTTTGCCGAGCCGCTGAAGCTGCGCAACGGCTCGTCCATTGCCGGCTAC
GACCTGATGGTCGAGACCTATGGCACGCTCAATGCGGCCCGCTCCAACGCGGTGCTGGTGTGCCACGCGCTCAATGCC
TCGCACCATGTGGCCGGCGTCTATGCGGAAGACCCGCGCGACGTGGGCTGGTGGGACAACATGGTCGGGCCCGGCAA
GCCGCTCGATACCAATCGCTTTTTCGTCATCGGCGTCAACAACCTGGGCTCGTGCTTCGGCTCGACCGGCCCGATGAGC
CTCAATCCGGCCACCGGCGCGCCCTACGGCGCAGCGTTCCCGGTGGTGACGGTGGAAGACTGGGTCAATGCGCAGGC
GCTCGTGGCCGACGCGTTCGGCATCACGCAGTTCGCCGCGGTGATGGGCGGCAGCCTGGGCGGCATGCAGGCGGTGG
CCTGGAGCCTGCTGTACCCGGAACGCCTGCGCCACTGCATCGTGGTCGCGTCCACGCCCAAGCTGTCGGCGCAGAATA
TCGCCTTCAACGAGGTCGCGCGCAGCGCCATCCTGTCGGACCCCGACTTCCACGGCGGCAACTACTACGCGCATGGCG
TCAAGCCCAAGCGCGGCCTGCGCGTGGCGCGCATGATCGGCCATATCACCTACCTGTCGGACGAGGACATGGCCGAGA
AATTCGGCCGCGAGCTGAAGACCGACGACATCCGCTTCTCCTTCGATGTCGAGTTCCAGGTAGAGAGCTACCTGCGCTA
CCAGGGCGACAAGTTCGCCGAATACTTCGACGCCAACACCTACCTGCTGATCACGCGCGCGCTCGACTACTTCGACCCG
GCGCTGGCCTTCGGCGGCGACCTGACGCGCGCGATGGCGCAGACCCAGGCCAGCTTCCTGGTGGCGAGCTTCGGCAC
CGACTGGCGCTTCGCGCCCAGCCGCAGCCGCGAGCTGGTCAAGGCGCTGCTGGACAACAAGCGCCCGGTCTCGTACG
CCGAGATCGATGCGCCGCACGGCCACGATGCCTTCCTGCTCGACGACCCGCGCTATCACAACCTGATGCGCGCCTACT
ACGACCGCATCGCAGAGGAGATCGGCGCATGA

- Amino acid sequence (with N-terminal His-tag)

MHHHHHHTDVALPTAAPAAFDLPPDSVGVVAPQRMHFAEPLKLRNGSSIAGYDLMVETYGTLNAARSNAVLVCHALNASHHV
AGVYAEDPRDVGWWDNMVGPGKPLDTNRFFVIGVNNLGSCFGSTGPMSLNPATGAPYGAAFPVVTVEDWVNAQALVADAF
GITQFAAVMGGSLGGMQAVAWSLLYPERLRHCIVVASTPKLSAQNIAFNEVARSAILSDPDFHGGNYYAHGVKPKRGLRVARM
IGHITYLSDEDMAEKFGRELKTDDIRFSFDVEFQVESYLRYQGDKFAEYFDANTYLLITRALDYFDPALAFGGDLTRAMAQTQAS
FLVASFGTDWRFAPSRSRELVKALLDNKRPVSYAEIDAPHGHDAFLLDDPRYHNLMRAYYDRIAEEIGA

3 Supplementary Figures

3.1 Biochemical characterization of *Cn*MetXs

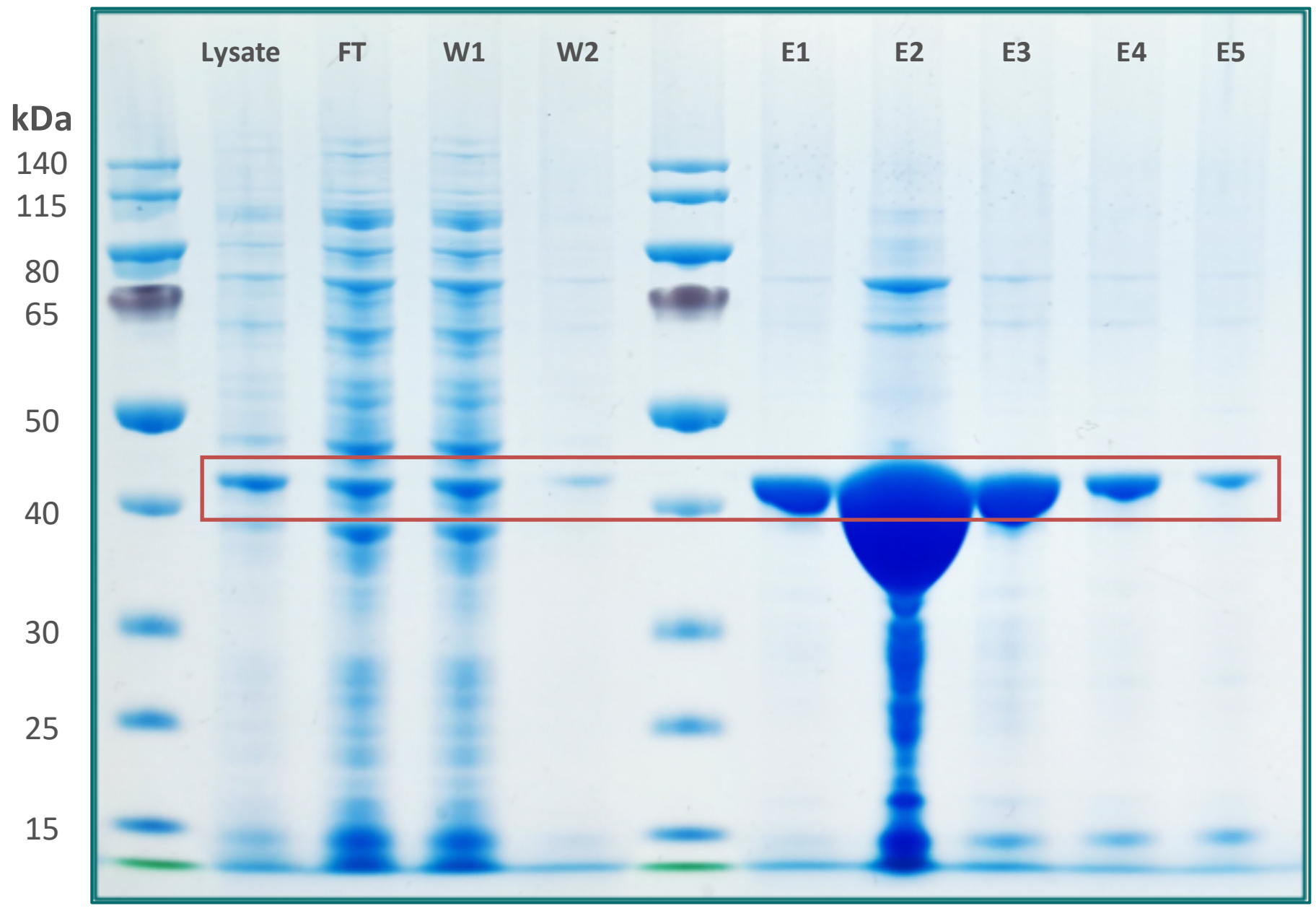

Figure S1. SDS-PAGE analysis of *Cn*MetXS purification. The molecular weight of 43 kDa corresponds to *Cn*MetXS. The fractions are shown as follows: Lysate: cell-free extract; FT: flowthrough; W1: 1st wash fraction; W2: 2nd wash fraction; E1-E5: collected elution fractions. Electrophoresis was performed for 2 h at 120 V and room temperature. Gels were stained overnight in a colloidal Coomassie staining solution and afterwards washed with water. PageRuler™ Prestained Protein Ladder, 10–180 kDa by Thermo Scientific, was used as a size standard marker (M).

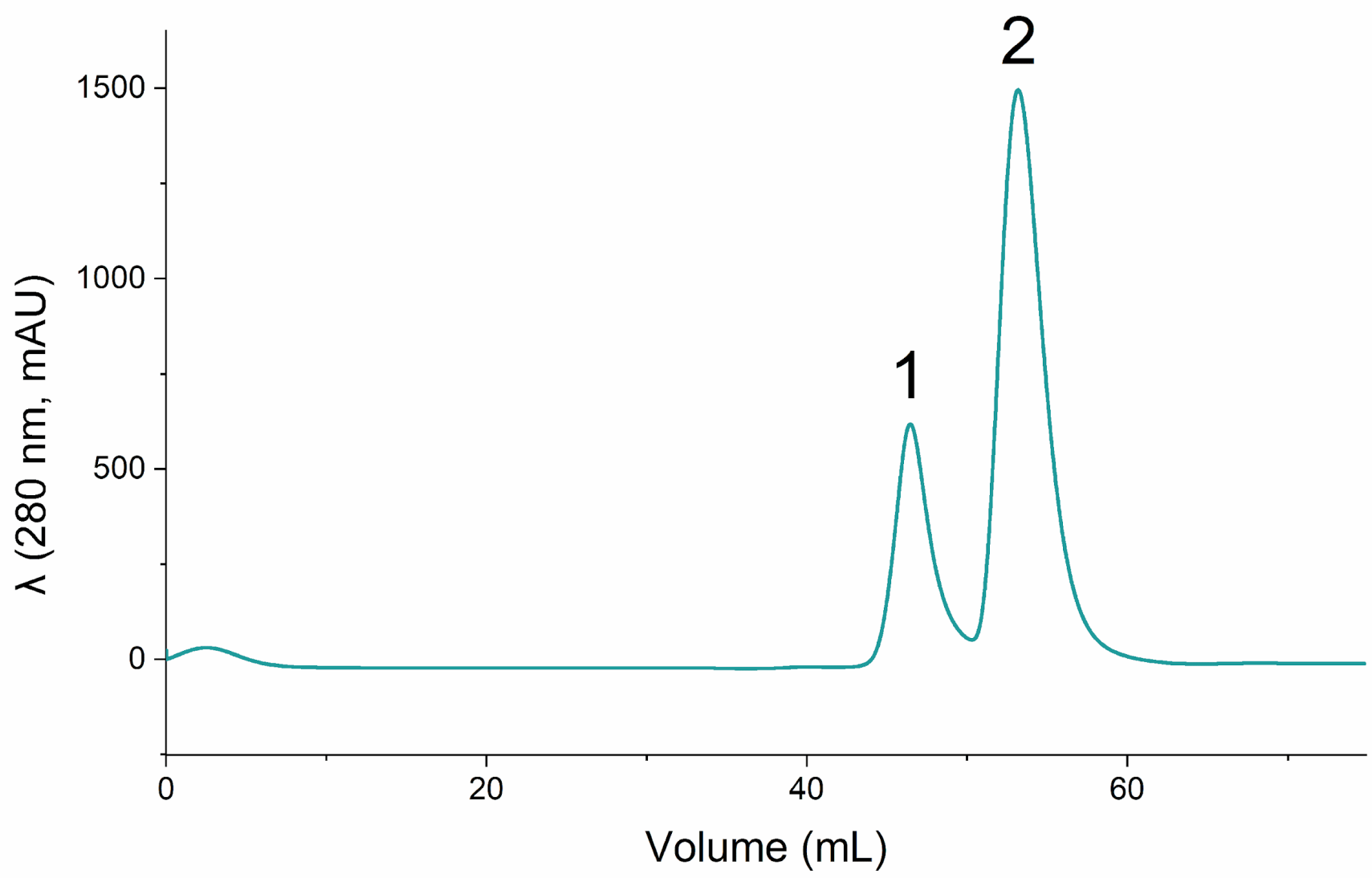

Figure S2. Size-exclusion chromatography (SEC) of *Cn*MetXS, most of the protein is present as a dimer (peak 2), some as a higher multimer (peak 1). Column: HiLoad™ 16/600 Superdex 75 pg. Conditions: 20 mM TRIS buffer with 150 mM NaCl, pH 7.5.

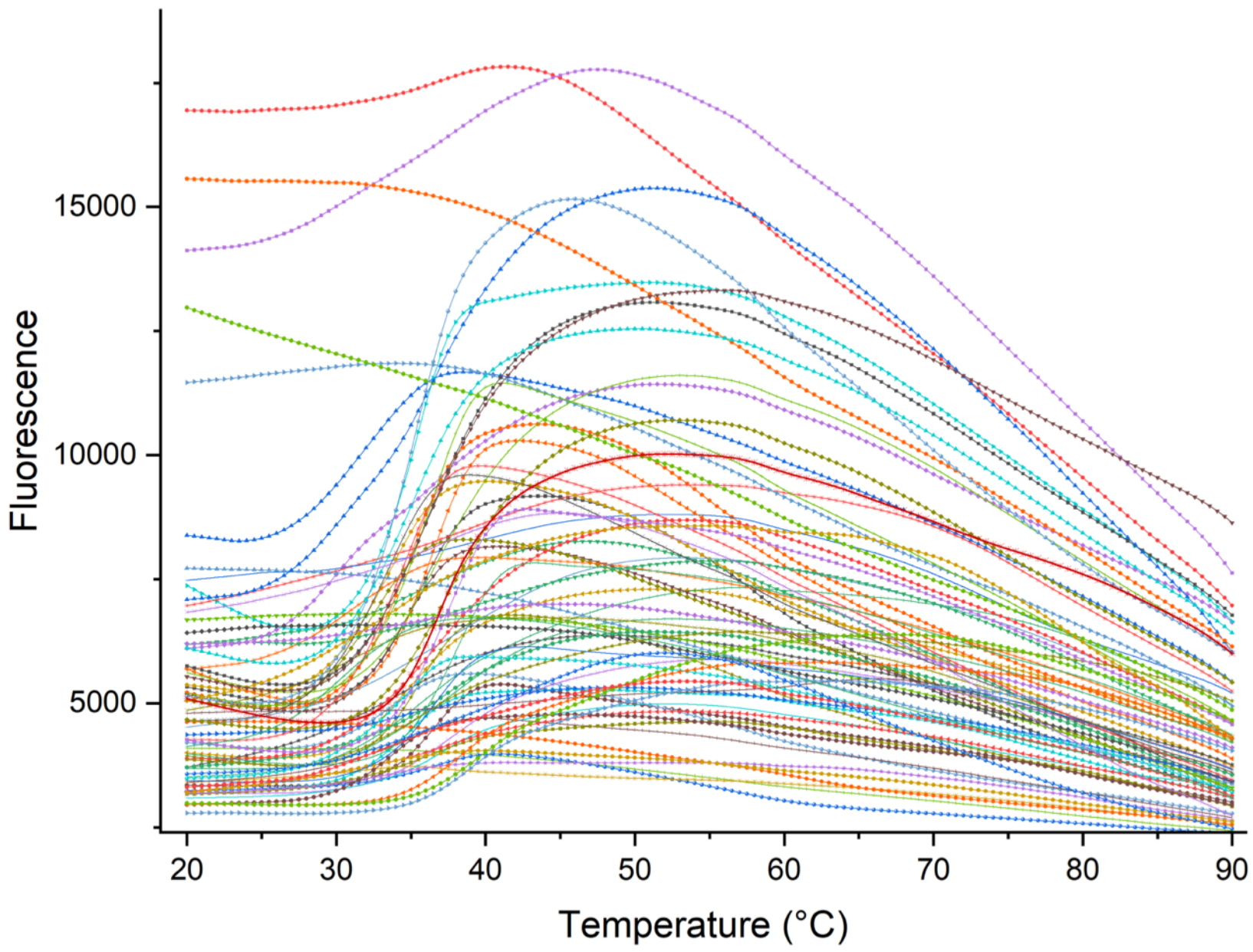

Figure S3. Result of the DSF assay of *Cn*MetXS using the dimeric fraction as isolated by SEC.

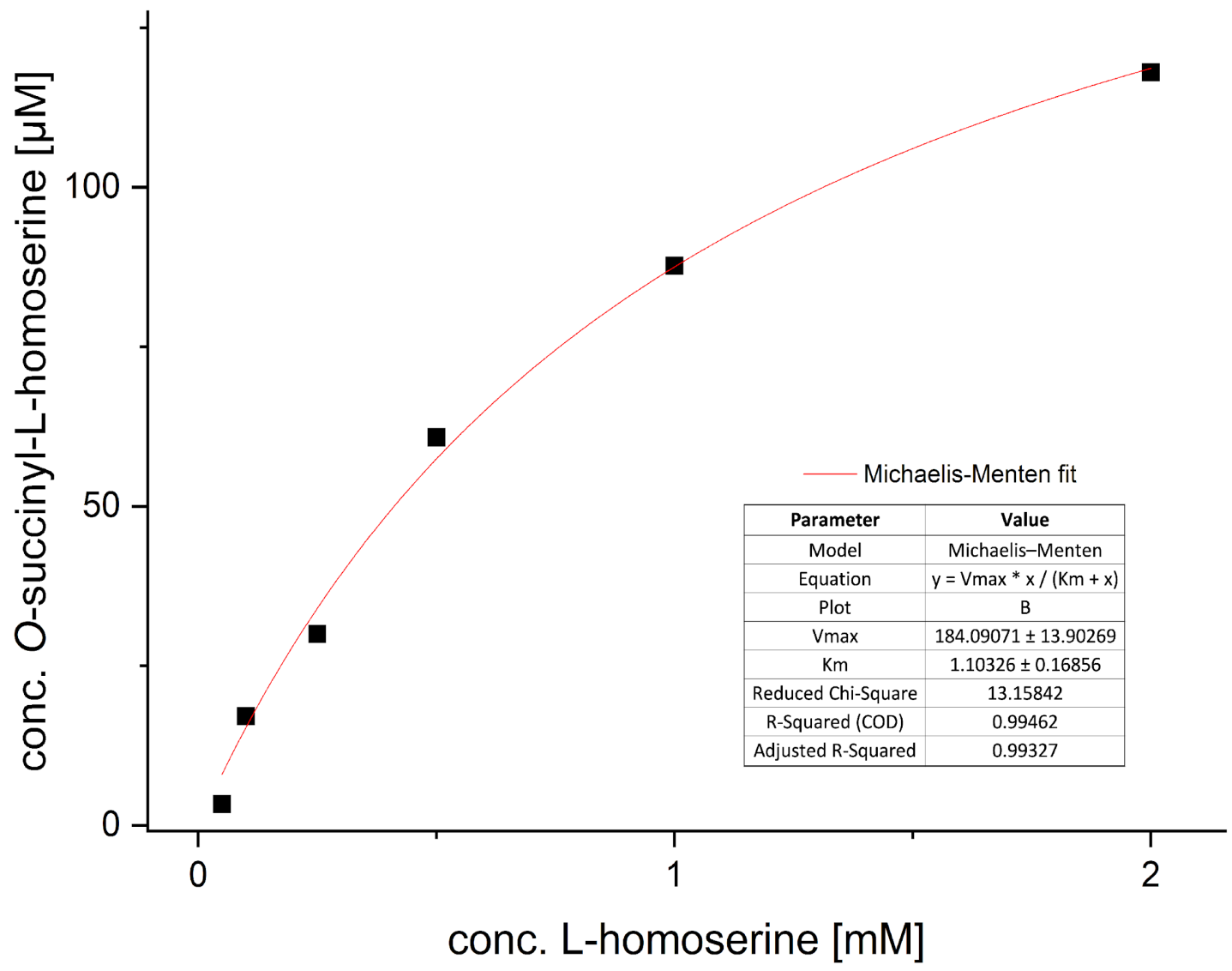

Figure S4. Kinetic studies of *Cn*MetXS towards L-homoserine in the presence of 1 mM succinyl-CoA. HPLC areas of *O*-succinyl-L-homoserine are shown. Mean 1.1 mM ± 0.17; n = 3. Non-linear regression: Michaelis-Menten function with an orthogonal distance regression using OriginPro2020 (OriginLab Corporation, Northampton, MA, USA). Reaction conditions: 20 mM KPi, 150 mM NaCl, pH 7.5, 30 °C.

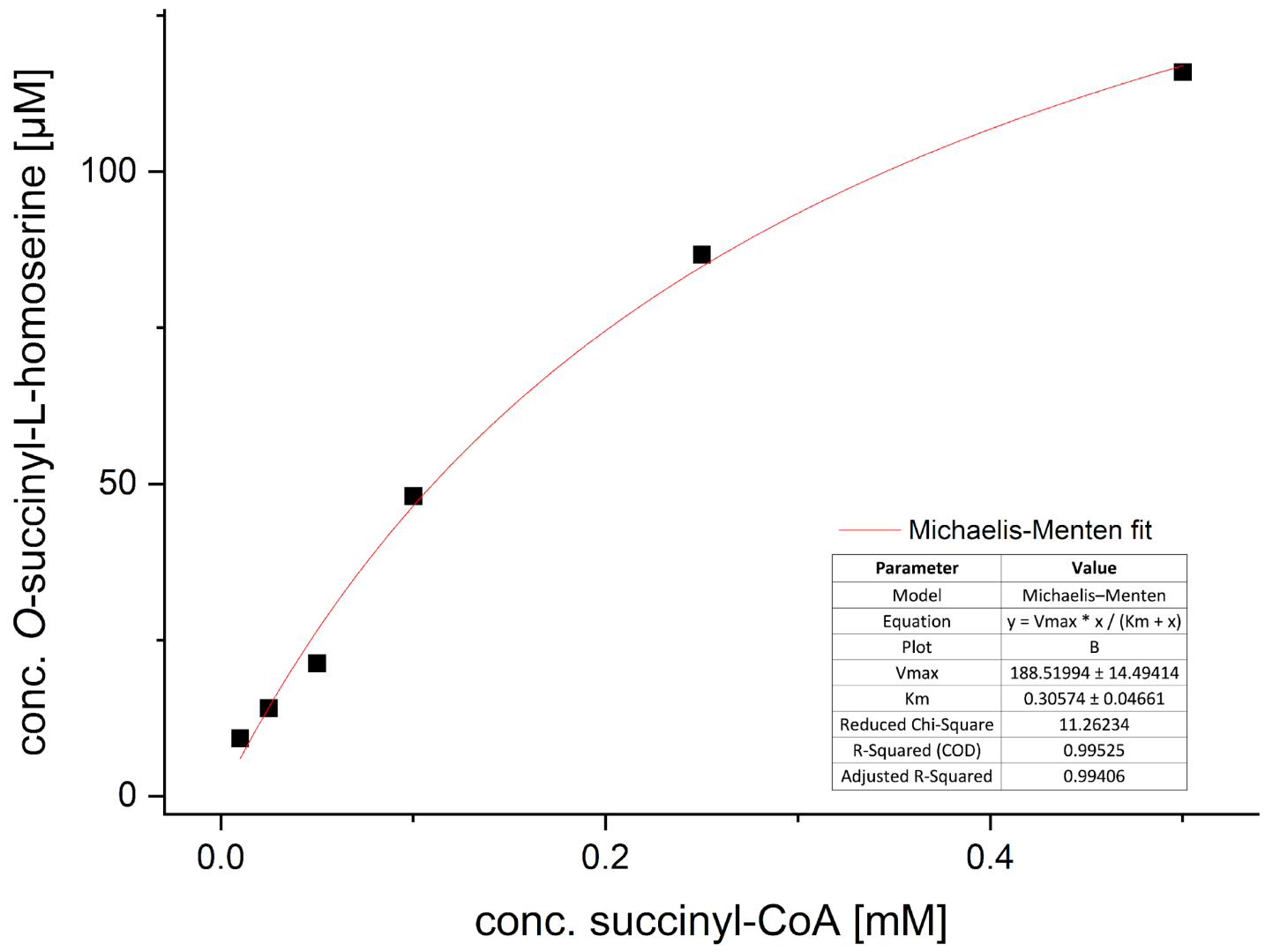

Figure S5. Kinetic studies of *Cn*MetXS towards succinyl-CoA in the presence of 3 mM L-homoserine. Concentrations of areas of *O*-succinyl-L-homoserine are shown. Mean 0.31 mM ± 0.05; n = 3. Non-linear regression: Michaelis-Menten function with an orthogonal distance regression using OriginPro2020 (OriginLab Corporation, Northampton, MA, USA). Reaction conditions: 20 mM KPi, 150 mM NaCl, pH 7.5, 30 °C.

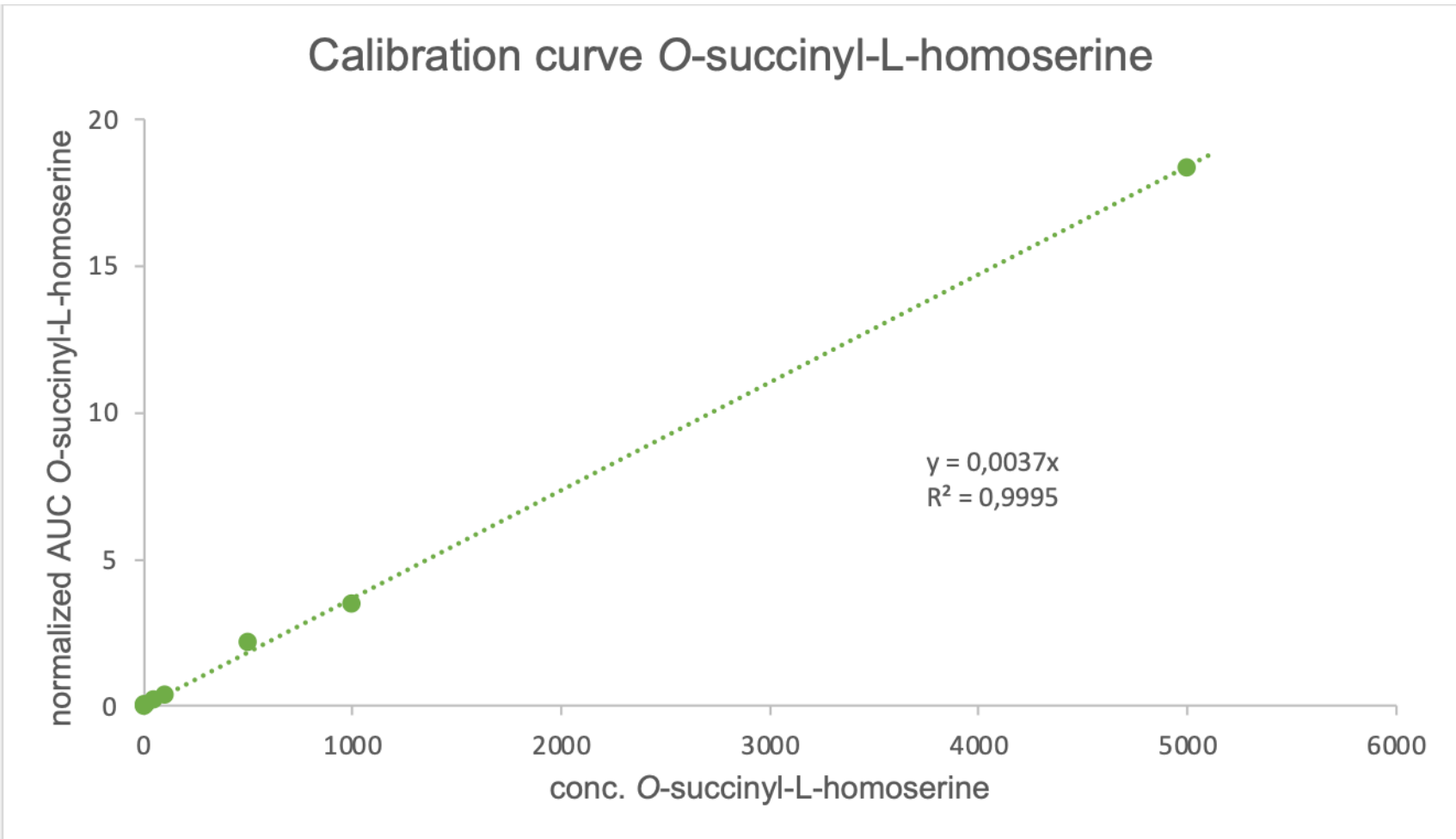

Figure S6. HPLC calibration curve of *O*-succinyl-L-homoserine used for kinetic studies. Formula: y = 0.037x, R^2 = 0.9995. Linear regression using OriginPro2020 (OriginLab Corporation, Northampton, MA, USA).

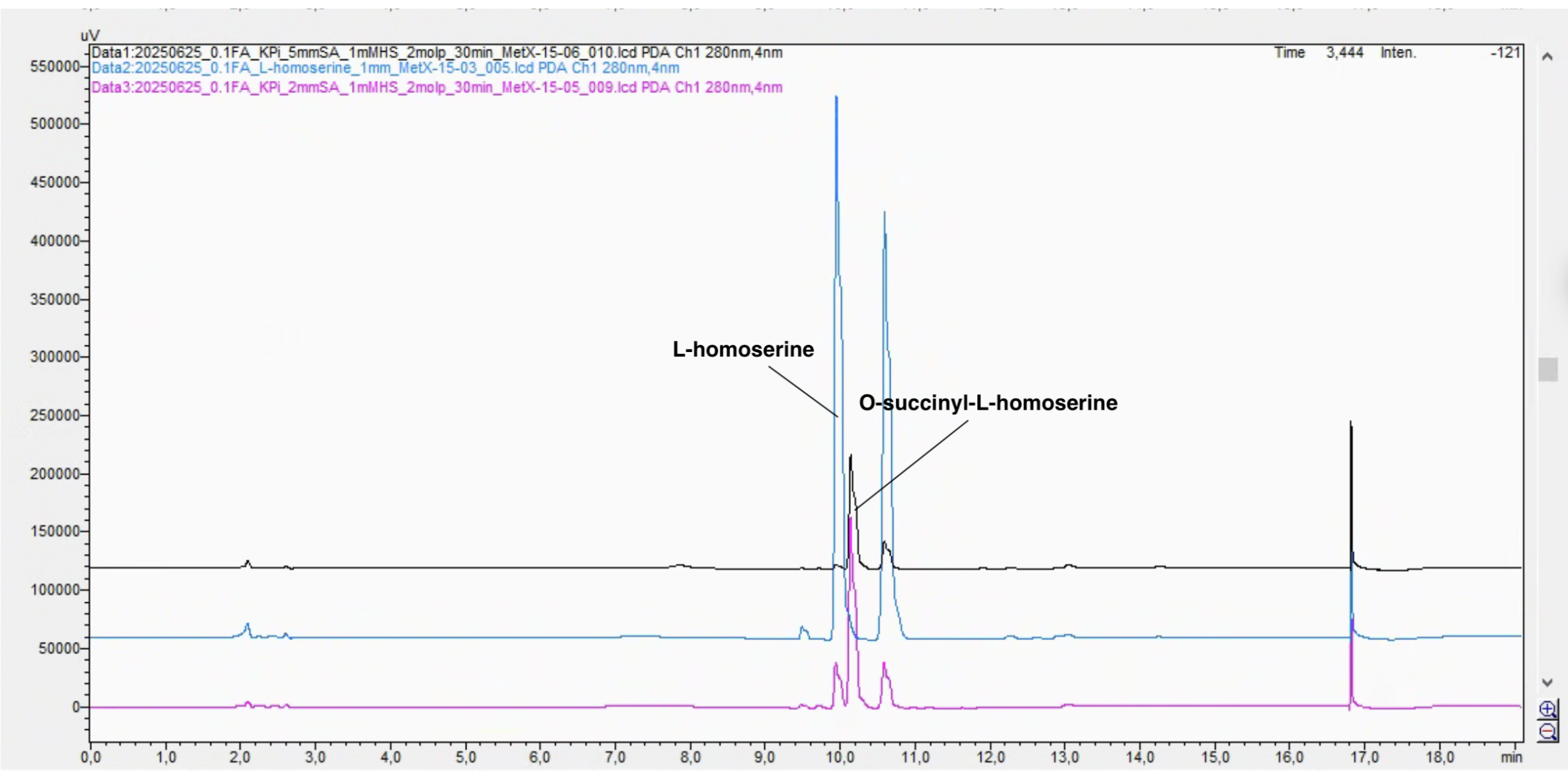

Figure S7. HPLC chromatogram of derivatized L-homoserine (blue) and reaction mixtures after derivatization (pink, black).

3.2 Structural analysis of *Cn*MetXS

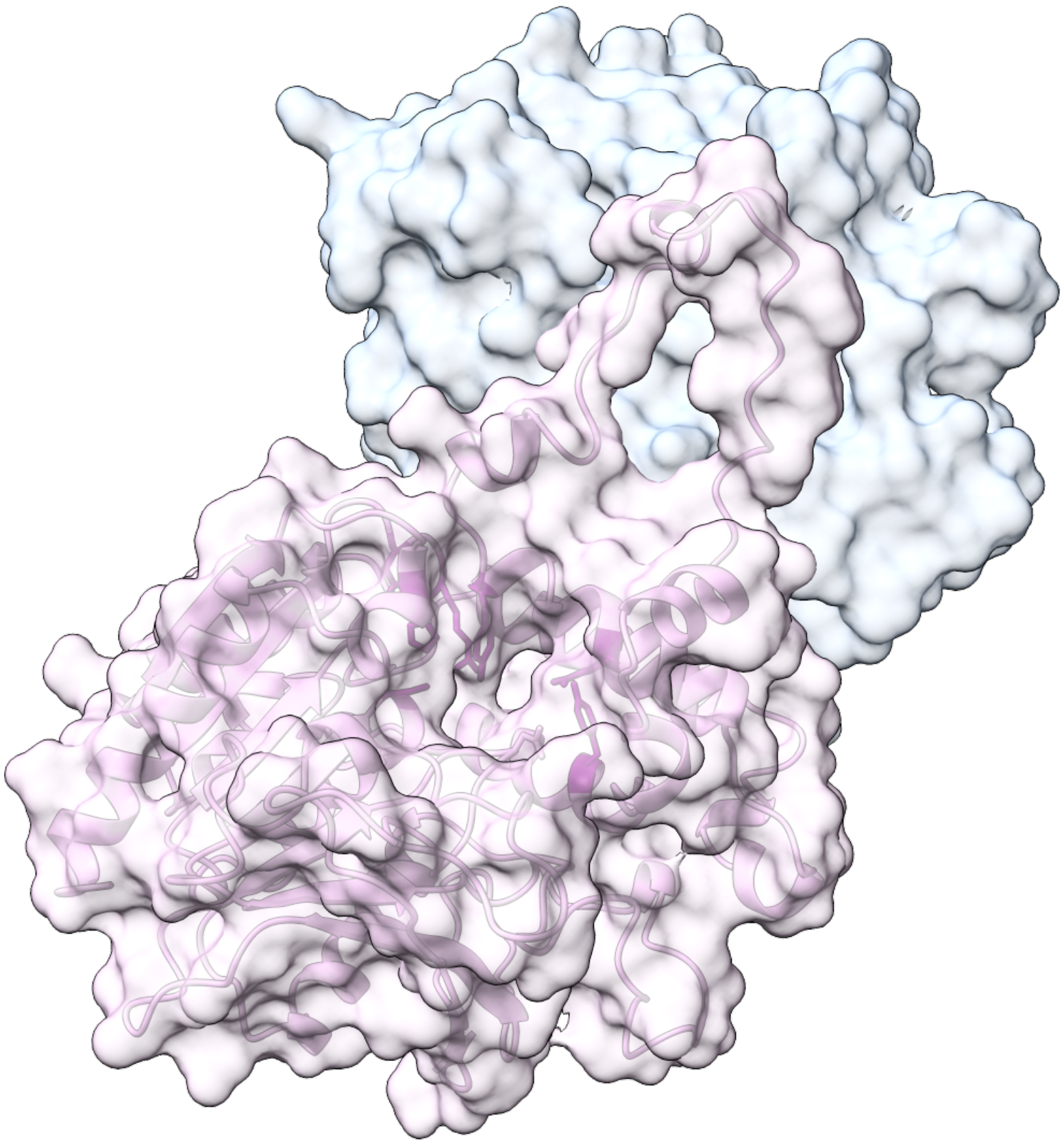

Figure S8. View on the active site of *Cn*MetXS, chain A.

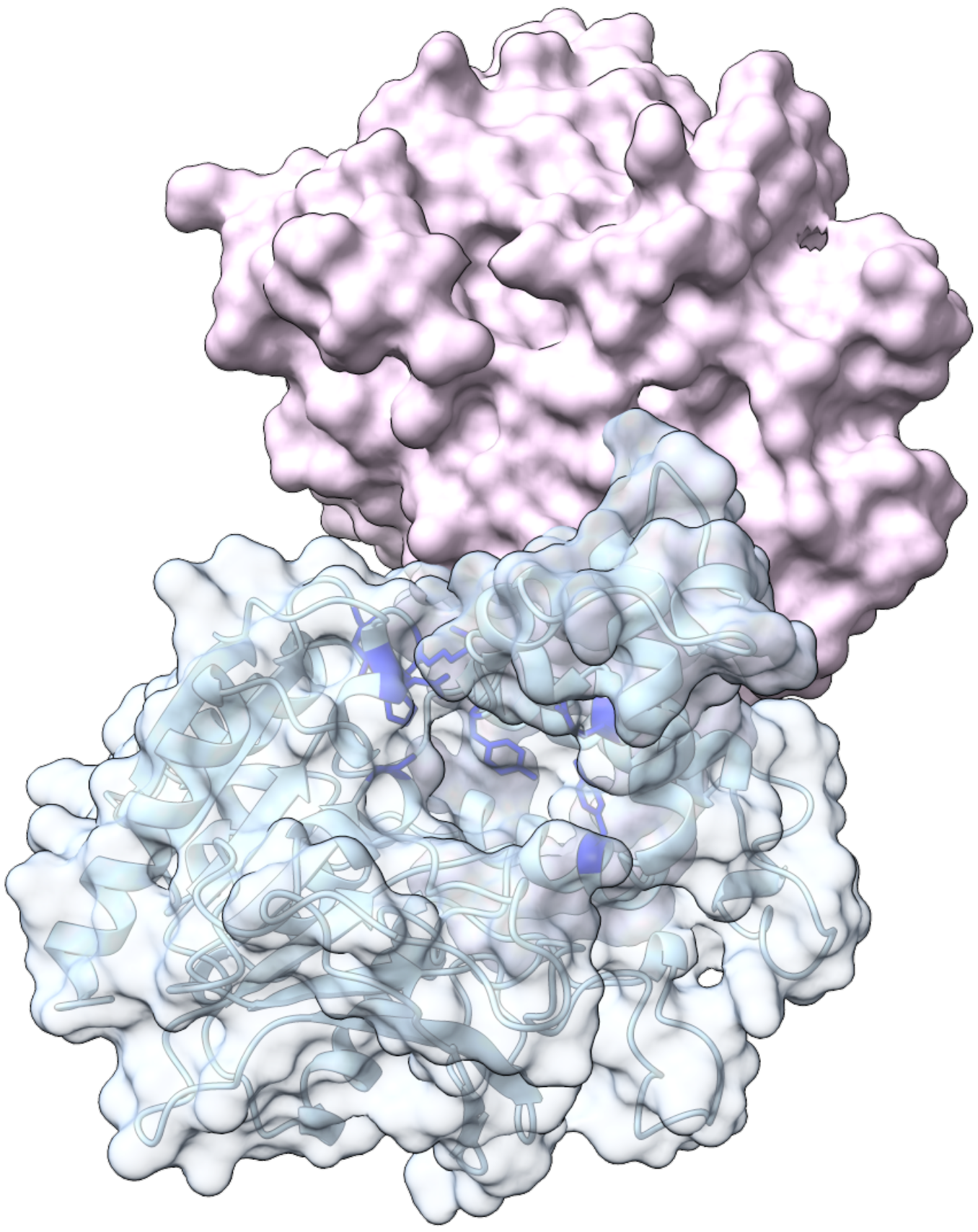

Figure S9. View on the active site of *Cn*MetXS, chain B.

3.2.1 Differences between the chain A and B in the crystal structure of *Cn*MetXS

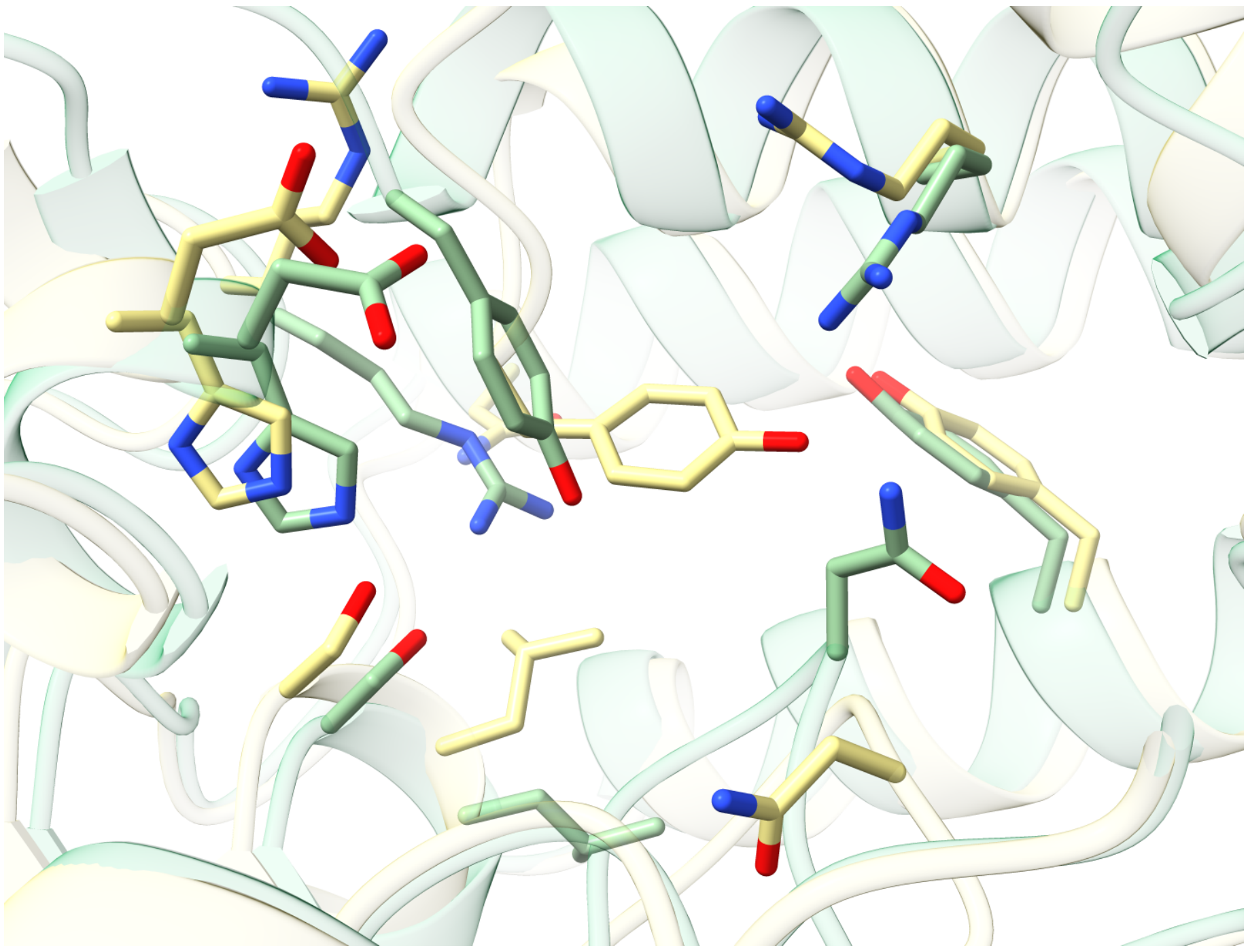

Figure S10. Zoomed in view of the active site, comparison of chain A (green) and B (yellow). Active site residues are shown as sicks.

3.2.2 Characterization of *Cn*MetXS as a member of the MetX-G2 group

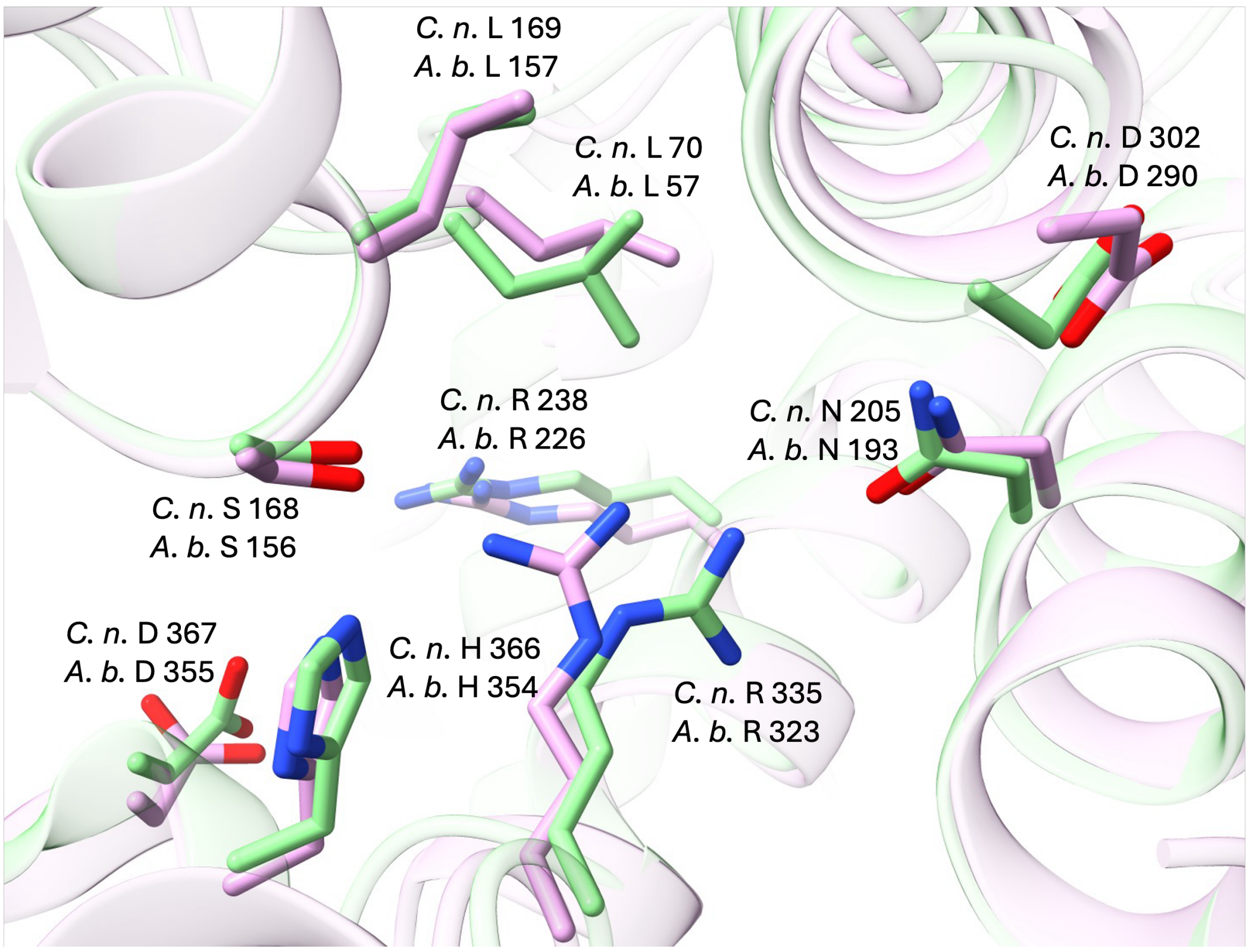

Figure S11. Comparison of active site residues of *Cn*MetXS (pink) and MetX from *Acinetobacter baylyi* (NCBI CR543861, green). Residues highlighted according to Bastard et al.[9] The residues found in *Cn*MetXS place the enzyme in the MetX-G2 group. *C. n.: C. necator, A. b.: A. baylyi.*

3.2.3 Structure of the conserved lid-domain and comparison to other structurally similar MetX-HATs

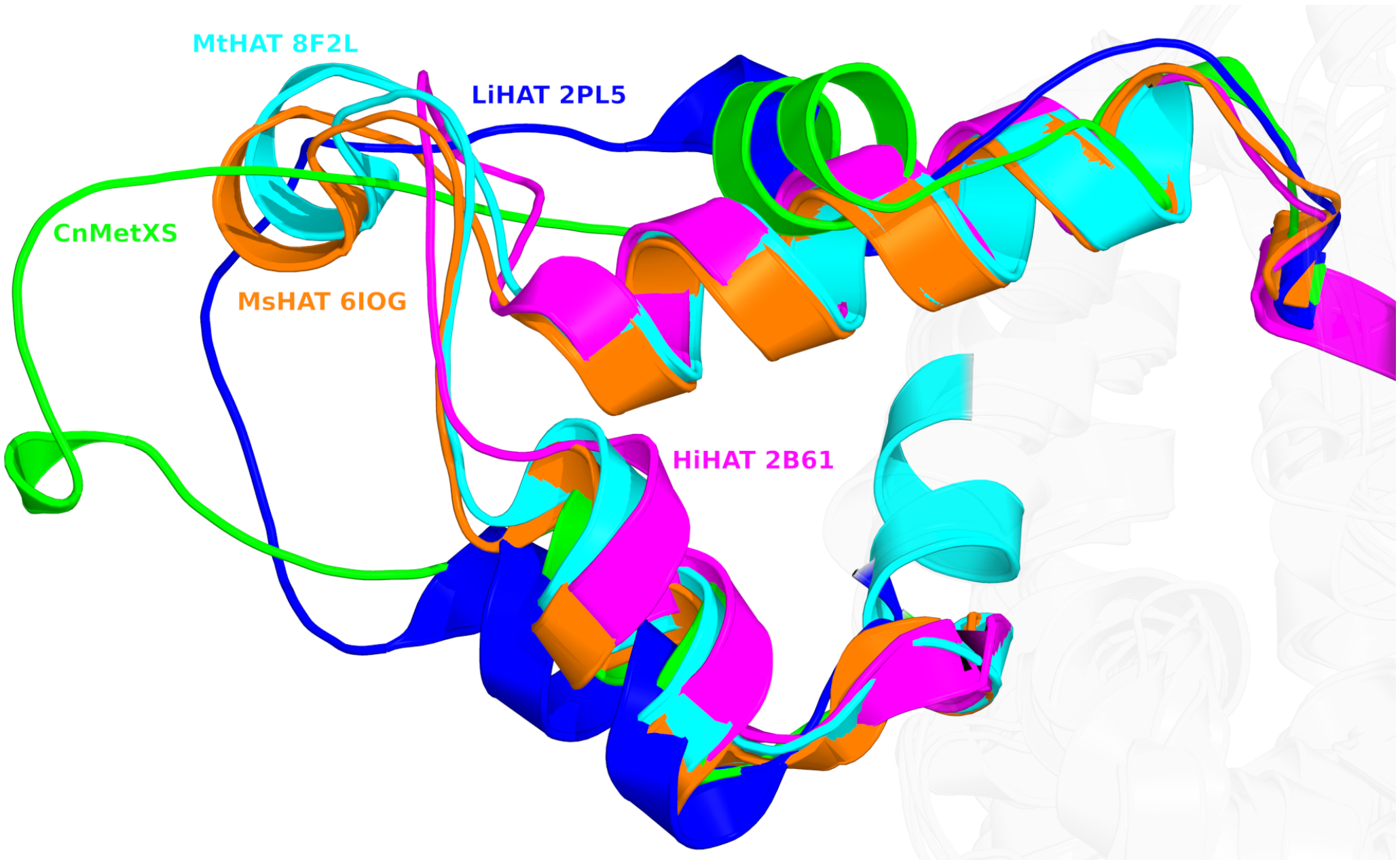

Figure S12. Structural comparison of the flexible lid region in *Cn*MetXS and representative HAT enzymes. The structures were superimposed and are shown as cartoon diagrams. The flexible lid segment corresponding to residues 244–291 in *Cn*MetXS is highlighted for *Cn*MetXS, MtHAT (8F2L), MsHAT (6IOG), LiHAT (2PL5), and HiHAT (2B61) in green, cyan, orange, blue, and magenta, respectively. The remaining protein structure is shown in transparent gray.

3. Supplementary Tables

Table S1. Tested conditions in the DSF assay.

Well	Condition	Additives	Well	Condition	Additives
A1	Water		E1	Water	
A2	100mM MMT buffer pH 4.0		E2	100mM Ammonium chloride	
A3	100mM MMT buffer pH 4.5		E3	100mM Potassium chloride	
A4	100mM MMT buffer pH 5.0		E4	100mM Sodium chloride	
A5	100mM MMT buffer pH 5.5		E5	100mM Lithium chloride	
A6	100mM MMT buffer pH 6.0		E6	100mM Magnesium chloride	
A7	100mM MMT buffer pH 6.5		E7	100mM Calcium chloride	
A8	100mM MMT buffer pH 7.0		E8	100mM Manganese chloride	
A9	100mM MMT buffer pH 7.5		E9	10mM Nickel chloride	
A10	100mM MMT buffer pH 8.0		E10	100mM Cobalt chloride	
A11	100mM MMT buffer pH 8.5		E11	10mM Iron (III) chloride	
A12	100mM MMT buffer pH 9.0		E12	10mM Zinc chloride	
B1	100mM Na-Citrate pH 4.0		F1	100mM Sodium citrate	
B2	100mM Na-Acetate pH 4.5		F2	100mM Sodium sulfate	
B3	100mM Na-Citrate pH 5.0		F3	100mM Sodium phosphate	
B4	100mM Na-Citrate pH 5.5		F4	100mM Sodium acetate	
B5	100mM Na-Phosphate pH 6.0		F5	100mM Sodium malonate	
B6	100mM MES pH 6.0		F6	100mM Sodium tartrate	
B7	100mM MES pH 6.5		F7	100mM Sodium formate	
B8	100mM HEPES pH 7.0		F8	100mM Sodium fluoride	
B9	100mM HEPES pH 7.5		F9	100mM Sodium bromide	
B10	100mM Imidazole pH 8.0		F10	100mM Sodium nitrate	
B11	100mM TRIS-HCl pH 8.0		F11	100mM Sodium iodide	
B12	100mM TRIS-HCl pH 8.5		F12	100mM Sodium thiocyanate	
C1	Water	250mM NaCl	G1	50mM NaCl	
C2	100mM MMT buffer pH 4.0	250mM NaCl	G2	100mM NaCl	
C3	100mM MMT buffer pH 4.5	250mM NaCl	G3	200mM NaCl	
C4	100mM MMT buffer pH 5.0	250mM NaCl	G4	400mM NaCl	
C5	100mM MMT buffer pH 5.5	250mM NaCl	G5	600mM NaCl	
C6	100mM MMT buffer pH 6.0	250mM NaCl	G6	1000mM NaCl	
C7	100mM MMT buffer pH 6.5	250mM NaCl	G7	5% Glycerol	
C8	100mM MMT buffer pH 7.0	250mM NaCl	G8	10% Glycerol	
C9	100mM MMT buffer pH 7.5	250mM NaCl	G9	20% Glycerol	
C10	100mM MMT buffer pH 8.0	250mM NaCl	G10	5% DMSO	
C11	100mM MMT buffer pH 8.5	250mM NaCl			
C12	100mM MMT buffer pH 9.0	250mM NaCl			
D1	100mM Na-Citrate pH 4.0	250mM NaCl			
D2	100mM Na-Acetate pH 4.5	250mM NaCl			
D3	100mM Na-Citrate pH 5.0	250mM NaCl			
D4	100mM Na-Citrate pH 5.5	250mM NaCl			
D5	100mM Na-Phosphate pH 6.0	250mM NaCl			
D6	100mM MES pH 6.0	250mM NaCl			
D7	100mM MES pH 6.5	250mM NaCl			
D8	100mM HEPES pH 7.0	250mM NaCl			
D9	100mM HEPES pH 7.5	250mM NaCl			
D10	100mM Imidazole pH 8.0	250mM NaCl			
D11	100mM TRIS-HCl pH 8.0	250mM NaCl			
D12	100mM TRIS-HCl pH 8.5	250mM NaCl			

Table S2: Summary of data collection and refinement statistics.

	***C. necator* Homoserine *O*-succinyltransferase (*Cn*MetXS)**
Wavelength	1.0332 Å
Resolution range	47.2 - 1.86 (1.90 - 1.86)
Space group	P 1 21 1
Unit cell	51.51 94.392 90.025<br>90 91.702 90
Total reflections	502631 (26748)
Unique reflections	71746 (4352)
Multiplicity	7.0 (6.1)
Completeness (%)	99.3 (97.8)
Mean I/sigma(I)	8.5 (0.6)
Wilson B-factor	25.35
R-merge	0.146 (2.868)
R-meas	0.172 (3.503)
R-pim	0.090 (1.924)
CC1/2	0.996 (0.339)
Reflections used in refinement	71487 (2733)
Reflections used for R-free	3538 (148)
R-work	0.1891
R-free	0.2231
Number of non-hydrogen atoms:	6492
macromolecules	5903
ligands	80
solvent	509
Protein residues	756
RMS(bonds)	0.006
RMS(angles)	0.96
Ramachandran favored (%)	98.14
Ramachandran allowed (%)	1.86
Ramachandran outliers (%)	0.00
Rotamer outliers (%)	0.17
Clash score	3.67
Average B-factor:	36.98
macromolecules	36.57
ligands	42.71
solvent	40.78

4. References

[1] W. Kabsch, "*XDS*" *Acta Crystallogr D Biol Crystallogr* **2010**, *66*, 125–132.
[2] P. R. Evans, G. N. Murshudov, "How good are my data and what is the resolution?" *Acta Crystallogr D Biol Crystallogr* **2013**, *69*, 1204–1214.
[3] A. J. McCoy, R. W. Grosse-Kunstleve, P. D. Adams, M. D. Winn, L. C. Storoni, R. J. Read, "*Phaser* crystallographic software" *J Appl Crystallogr* **2007**, *40*, 658–674.
[4] J. Jumper, R. Evans, A. Pritzel, T. Green, M. Figurnov, O. Ronneberger, K. Tunyasuvunakool, R. Bates, A. Žídek, A. Potapenko, A. Bridgland, C. Meyer, S. A. A. Kohl, A. J. Ballard, A. Cowie, B. Romera-Paredes, S. Nikolov, R. Jain, J. Adler, T. Back, S. Petersen, D. Reiman, E. Clancy, M. Zielinski, M. Steinegger, M. Pacholska, T. Berghammer, S. Bodenstein, D. Silver, O. Vinyals, A. W. Senior, K. Kavukcuoglu, P. Kohli, D. Hassabis, "Highly accurate protein structure prediction with AlphaFold" *Nature* **2021**, *596*, 583–589.
[5] M. Varadi, S. Anyango, M. Deshpande, S. Nair, C. Natassia, G. Yordanova, D. Yuan, O. Stroe, G. Wood, A. Laydon, A. Žídek, T. Green, K. Tunyasuvunakool, S. Petersen, J. Jumper, E. Clancy, R. Green, A. Vora, M. Lutfi, M. Figurnov, A. Cowie, N. Hobbs, P. Kohli, G. Kleywegt, E. Birney, D. Hassabis, S. Velankar, "AlphaFold Protein Structure Database: massively expanding the structural coverage of protein-sequence space with high-accuracy models" *Nucleic Acids Research* **2022**, *50*, D439–D444.
[6] M. Varadi, D. Bertoni, P. Magana, U. Paramval, I. Pidruchna, M. Radhakrishnan, M. Tsenkov, S. Nair, M. Mirdita, J. Yeo, O. Kovalevskiy, K. Tunyasuvunakool, A. Laydon, A. Žídek, H. Tomlinson, D. Hariharan, J. Abrahamson, T. Green, J. Jumper, E. Birney, M. Steinegger, D. Hassabis, S. Velankar, "AlphaFold Protein Structure Database in 2024: providing structure coverage for over 214 million protein sequences" *Nucleic Acids Research* **2024**, *52*, D368–D375.
[7] P. Emsley, K. Cowtan, "*Coot* : model-building tools for molecular graphics" *Acta Crystallogr D Biol Crystallogr* **2004**, *60*, 2126–2132.
[8] D. Liebschner, P. V. Afonine, M. L. Baker, G. Bunkóczi, V. B. Chen, T. I. Croll, B. Hintze, L.-W. Hung, S. Jain, A. J. McCoy, N. W. Moriarty, R. D. Oeffner, B. K. Poon, M. G. Prisant, R. J. Read, J. S. Richardson, D. C. Richardson, M. D. Sammito, O. V. Sobolev, D. H. Stockwell, T. C. Terwilliger, A. G. Urzhumtsev, L. L. Videau, C. J. Williams, P. D. Adams, "Macromolecular structure determination using X-rays, neutrons and electrons: recent developments in *Phenix*" *Acta Crystallogr D Struct Biol* **2019**, *75*, 861–877.
[9] K. Bastard, A. Perret, A. Mariage, T. Bessonnet, A. Pinet-Turpault, J.-L. Petit, E. Darii, P. Bazire, C. Vergne-Vaxelaire, C. Brewee, A. Debard, V. Pellouin, M. Besnard-Gonnet, F. Artiguenave, C. Médigue, D. Vallenet, A. Danchin, A. Zaparucha, J. Weissenbach, M. Salanoubat, V. De Berardinis, "Parallel evolution of non-homologous isofunctional enzymes in methionine biosynthesis" *Nat Chem Biol* **2017**, *13*, 858–866.
